# Identification of a potent NAFLD drug candidate for controlling T2DM-mediated inflammation and secondary damage *in vitro* and *in vivo*

**DOI:** 10.3389/fphar.2022.943879

**Published:** 2022-08-19

**Authors:** Md Samsuzzaman, Jae Hyuk Lee, Hyejin Moon, Jisue Lee, Heaji Lee, Yunsook Lim, Myoung Gyu Park, Hakwon Kim, Sun Yeou Kim

**Affiliations:** ^1^ College of Pharmacy, Gachon University, Incheon, South Korea; ^2^ Department of Applied Chemistry and Global Center for Pharmaceutical Ingredient Materials, Kyung Hee University, Seoul, Gyeonggi, South Korea; ^3^ Department of Food and Nutrition, Kyung Hee University, Seoul, South Korea; ^4^ MetaCen Therapeutics Company, Suwon, Gyeonggi, South Korea; ^5^ Gachon Institute of Pharmaceutical Science, Gachon University, Incheon, South Korea

**Keywords:** glucotoxicity, NASH, KHAG-04, inflammation, lipogenesis

## Abstract

Accumulation of glucose/sugar results in the formation of reactive di-carbonyl compounds such as MGO and GO that interact with several amino acids and proteins to form toxic advanced glycation end products (AGEs). Induction of AGEs breakdown can control symptoms and severity in T2DM and other related complications like NAFLD where AGEs are the key players. Therefore, an AGE cross-link breaker has been suggested for preventing the onset/progression of NAFLD. In this study, we reported novel synthetic naphthalene-2-acyl thiazolium derivatives (KHAGs). Among synthesized KHAG derivatives, we observed that a novel KHAG-04, a 1,4-dimethoxynaphthalen-2-acyl thiazolium salt which is an analog of alagebrium, dramatically cleaves MGO/GO-AGE cross-links, and it also inhibited inflammation by lowering the level of nitric oxide production and IL-1β and TNF-α secretion in LPS and/or MGO-AGE–activated macrophage. Moreover, it also reduced FFA and MGO-AGE–induced lipogenesis in Hep-G2 cells. In mice, KHAG-04 significantly reduced the level of glyoxal in the liver, which was induced by DMC. Furthermore, KHAG-04 treatment significantly reduced blood glucose levels, lipid accumulation, and inflammation in the NAFLD/T2DM animal model. Novel KHAG-04–mediated induction of AGEs breakdown could be the possible reason for its anti-inflammatory, antihyperglycemic, and anti-lipidemic effects in cells and NAFLD in the T2DM animal model, respectively. Further research might explore the pharmacological efficacy and usefulness and consider the ability of this compound in the treatment strategy against various models of NAFLD in T2DM where MGO/GO-AGEs play a key role in the pathogenesis.

## Introduction

Type 2 diabetes mellitus (T2DM) is a metabolic disorder with high glucose levels in the blood (American Diabetes Association, 2009). A high blood glucose level condition is called hyperglycemia. Hyperglycemia is the root of the secondary damage or complications to other vital organs such as hepatic, cardiovascular, renal, retinopathy, and neuronal system (Lin et al., 2005; Targher et al., 2005; Rask-Madsen and King, 2013). Low glucose metabolism and increased levels of glucose/sugar induce a condition where glycation or oxidation of glucose products results in the formation of reactive metabolites such as methylglyoxal (MGO) and glyoxal (GO) (Singh et al., 2014; Brings et al., 2017). Highly reactive MGO and GO can interact with several proteins in the body resulting in the formation of advanced glycation end products (AGEs) (Singh et al., 2014). Increased accumulation of AGEs is another characteristic feature of T2DM (Vlassara and Uribarri, 2014). Several previous studies have highlighted a number of treatment strategies in practice for the treatment of T2DM by lowering the blood glucose level in patients. However, the treatment strategies are not fully compliant and successful for the patients because of several adverse effects (Marín-Peñalver et al., 2016). Interestingly, AGEs formed by several glucose oxidations or glycation products are reported to be responsible for secondary damage or health complications. Therefore, inhibition of total AGEs levels either by inhibiting formation or inducing breakdown could be a better alternative target for the management of T2DM-related organ injury and complications.

MGO/GO-AGEs were reported to be responsible for microglial as well as macrophage activation resulting in neuronal as well as systemic inflammation (Subedi et al., 2020). Inflammation and complications in other organs such as the kidney, heart, and liver fall under systemic inflammation. Several disease conditions such as AD, PD, cognitive decline, retinopathy, and stroke are T2DM-related neuroinflammatory complications, while cardiovascular, renal complications, and NAFLD are examples of T2DM-mediated systemic inflammation (Cheung et al., 2007; Laakso, 2010; Lin and Sun, 2013; Chung et al., 2015; Cholerton et al., 2016; Rosato et al., 2019). Studies suggested that almost 70–87% of T2DM animals or patients are suffering from non-alcoholic fatty liver disease (NAFLD) (Prashanth et al., 2009; Dharmalingam et al., 2018). The major symptomatic features of NAFLD are steatosis, non-alcoholic steatohepatitis (NASH), fibrosis, cirrhosis, and hepatocellular carcinoma (Dharmalingam et al., 2018). Increased glucose accumulation and lipid deposition in the body can cause induction of MGO and GO levels that ultimately increase the formation of MGO/GO-AGEs by the process of glycation and autooxidation. All these conditions are directly and indirectly involved in the activation of the immune cells, especially macrophages. Overactivation of macrophages is a well-known condition for secondary damage to organs such as the liver in NAFLD (Kmieć, 2001; Fujiwara and Kobayashi, 2005). Hence, NAFLD is a major complication of T2DM and increased glycation products, and dicarbonyl compound–derived AGEs (MGO/GO-AGEs) are the key factor responsible for this condition. Thus, inhibition of the level of MGO/GO or induction of MGO-GO-AGEs breakdown might help lower fat/lipid accumulation and inflammation by reducing macrophage activation.

**FIGURE 1:**
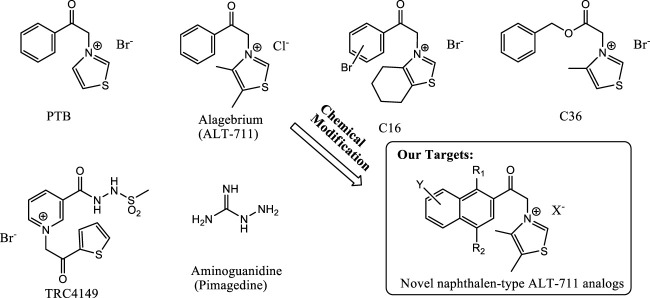
Structures of previously known AGEs breakers and our target molecules. The structure of PTB, alagebrium, C16, C36, TRC4149, aminoguanidine, ALT-711, and ALT-711 analog are presented here for further understanding of KHAG series compounds synthesis.

AGEs cross-links were believed to be irreversible until the first study about alagebrium (ALT-711) was reported as a novel AGEs breaker that cleaved the linkage molecule and released the free amines. This potent compound was useful in controlling blood pressure in heart failure patients by breaking AGE-mediated blood vessel stiffening. ALT-711 was also beneficial in the treatment of T1DM in rat models (Cheng et al., 2007). The pharmacological efficacy of this drug raises a shade in the utilization of this candidate for human use. In addition to ALT-711, several other candidates such as pyridinium analogs TRC4149 and 3-(2-benzyloxy)-2-oxoethyl-4-methylthiazol-3-ium bromide (C36) are also still under investigation for their AGE breaking effect and subsequent benefits against T2DM-related complications (Figure 1). Reactive glycation end products can interact with different proteins resulting in different AGE types; hence, it is very challenging to find AGE breakers. However, MGO-derived AGEs are reported to have several organ toxicities (Goudarzi et al., 2018). Despite various reports on MGO-AGE–mediated toxicity in different organs, its role in the liver system is yet to be reported. By using LC-MS/MS, it was found that increased MG and MGO-AGEs formation in hepatic steatosis *in vivo* may be localized to the liver (Maldonado et al., 2015). Previous studies focused on the pathophysiology of NAFLD with special emphasis on the role of AGEs in NAFLD progression to non-alcoholic steatohepatitis and liver fibrosis. Moreover, manipulation to reduce AGEs content in the therapies targeting the AGEs/RAGE pathway on disease progression is also important for NAFLA/NASH therapy (Fernando et al., 2019). Finally, the driving of NAFLD by MGO-AGEs may increase the progression of liver fibrosis. MGO and GO are the key precursors for toxic AGEs formation. Therefore, targeting MGO/GO-AGEs can be highly useful for the treatment of AGE-mediated complications in T2DM.

Interestingly, we found a novel skeleton for an MGO-AGE breaker from a natural resource and established in-house small-molecule libraries to serve as a more powerful MGO-AGE breaker for the bioactive small-molecule discovery of NAFLD. In this study, we also synthesized various naphthalene-type derivatives containing an acyl thiazolium moiety in the structure of ALT-711, a well-known AGE cross-links breaker, to develop a candidate compound for AGEs breakdown. This study focuses on the pathophysiology of NAFLD with special emphasis on the role of potential MGO/GO-AGE breaker in NAFLD progression to NASH and liver fibrosis. This study aims to inhibit AGE toxicity *in vitro* and *in vivo* by lowering the AGEs level following cleaved AGE cross-links through treatment of a novel MGO-AGE breaker derived from our in-house chemical libraries including natural products.

## Materials and methods

### Chemicals and instruments

All chemical reagents used for synthesis were purchased from Acros Organics (United States), Alfa Aesar (United States), Sigma-Aldrich (United States), and Tokyo Chemical Industry (Japan) and were used without further purification. Solvents including acetonitrile (ACN), ethyl acetate (EA), dichloromethane (DMC), dimethyl sulfoxide (DMSO), and dimethylformamide (DMF) were used as received from TCI (Japan), Sigma-Aldrich (United States), Samchun Chemical Co., Ltd. (Korea), and Duksan Pure Chemicals Co., Ltd (Korea). 2-(Bromoacetyl)-naphthalene (1a) was purchased from Alfa Aesar (United States), and other starting compounds were prepared according to literatures: 1 b from 2-acetyl-1-naphthol (Zhang et al., 2010); 2a, 2b, 6a, 6b, and 6c from 1,4-naphthoquinone (Lee et al., 2021); and 2c and 4 from menadione (Jung et al., 2005; Kim et al., 2020). The reactions were monitored by thin-layer chromatography (TLC, silica gel 60 F_254_, Merck), and column chromatography was performed using silica gel 60 (particle size 0.040–0.063 mm, Merck). The ^1^H NMR and ^13^C NMR spectra were recorded on a Jeol 300 MHz spectrometer and Jeol 75 MHz spectrometer (Japan), respectively. Spectra are referenced relative to the chemical shift of tetramethylsilane (TMS). The melting points (m.p) were determined on a Barnsteand Electrothermal 9100 instrument (United Kingdom), and mass spectrometry (MS) spectra were recorded using Agilent 1260 Infinity models (United States).

### Synthesis of KHAG series

The detail synthesis methods of KHAG-04 compounds are provided in the supplementary materials.

### Cell culture

Raw 264.7 and HepG2 cells were purchased from the Korean Cell Line Bank (Seoul, Korea). Raw 264.7 cells were cultured in Dulbecco’s modified Eagle’s medium (DMEM) and HepG2 cells were cultured in RPMI-1640 medium, and both the cell culture medium were maintained, supplemented with 10% of fetal bovine serum (FBS) and 1% of penicillin/streptomycin, at 37°C in a humidified incubator containing 5% CO_2_.

### Cell viability assay

Cell viability was measured using the methyl thiazolyl blue tetrazolium bromide (MTT) assay. Raw 264.7 and HepG2 cells were seeded in 96-well plates. After incubation, cells were treated with several concentrations (0.1, 1, and 10 μM) of KHAG-04 for 24 h. After incubation for 24 h, MTT (0.5 mg/ml) solution was added to each well and the cells were incubated for 1 h. Then the medium was carefully suctioned, and DMSO was added to each well. The absorbance was taken at 570 nm using a microplate reader (Molecular Devices, CA, United States).

### NO assay

Raw 264.7 cells were seeded in 96-well plates and incubated for 24 h at 37°C in 5% CO_2_. After incubation for 24 h, cells were pretreated with several concentrations (0.1, 1, and 10 μM) of KHAG-04 for 30 min, followed by treatment with 100 ng/ml LPS and MGO-AGEs (2 mg/ml) for 24 h. After the conditioned medium was carefully transferred to 96-well plates, an equal volume of Gries reagent was added to each well. Then the nitrite concentration was quantified by evaluating the absorbance at 540 nm in the microplate reader (Molecular Devices, CA, United States).

### ELISA assay

IL-1β, IL-6, and TNF-α secretion were evaluated using enzyme-linked immunosorbent assay (ELISA), according to the manufacturer’s protocol. Raw 264.7 cells were seeded in 24-well plates and incubated for 24 h at 37°C in 5% CO_2_. After incubation, cells were pretreated with several concentrations (0.1, 1, and 10 μM) of KHAG-04 and KHAG-05 for 30 min, followed by treatment with 100 ng/ml LPS for 24 h; the conditioned medium was collected and centrifuged. Then IL-1β, IL-6, and TNF-α secretion were quantified using the supernatants *via* the respective ELISA kit (R&D Systems, MN, United States).

### Oil red O staining

HepG2 cells were seeded in a 35-mm dish and incubated for 24 h at 37°C in 5% CO_2_. After incubation, cells were pretreated with several concentrations (1 and 10 μM) of KHAG-04 for 1 h, followed by treatment with palmitic acid (0.5 mM), LPS (1 μg/ml), and MGO-AGEs (2 mg/ml) for 24 h. Then cells were fixed in 10% neutral buffered formalin (pH 6.8–7.2) for 1 h, washed with 60% isopropanol, and dried. Next, cells were stained with 0.5% oil red O solution for 20 min at 25°C in the shaker and washed with distilled water. After washing, the stained cells were observed under a Nikon Eclipse 80i microscope (Nikon, Tokyo, Japan) at ×100 magnification. Lipid droplets’ accumulation was quantified *via* measuring the absorbance at 510 nm in the microplate reader (Molecular Devices, CA, United States).

### Preparation of AGEs

Advanced glycation end products were prepared, following the procedure of Subedi et al. (2020), by incubating 5 mg/ml of BSA and 0.02% sodium azide (pH 7.4) with 10 mM of MGO/GO, which was diluted in PBS (1X), at 37 °C for 7 days. Samples were evaporated, filtered, and dialyzed by ZebaTM Spin Desalting Columns, 7K MWCO, 5 ml (Thermo Fisher Scientific, Waltham, MA), before further drying in a freeze dryer.

### AGE breaker assay

A TNBSA (2,4,6-trinitrobenzene sulfonic acid) assay was quantified to validate the effect of 13 compounds on MGO-AGEs breakdown, as described previously by Furlani et al. (2015), with slight modifications. In brief, 1 mg/ml GO-AGEs or MGO-AGEs was mixed with several concentrations (0.1 and 0.4 mM) of KHAG-04 or 15 compounds and incubated for 24 h at 37°C. After 24 h, TNBSA and NaHCO3 were added, and the ep-tubes were incubated for 2 h at 37°C. The chemical reaction was stopped by adding 10% SDS solution and 1M HCl to each ep-tube. Free amines were quantified by evaluating the absorbance at 335 nm in the microplate reader (Molecular Devices, CA, United States).

### Western blot

Raw 264.7 cells and hepatic tissue were harvested and homogenized with PRO-PREPTM protein extraction solution (iNtRON, Seoul, Korea) containing protease inhibitors. The cell and tissue lysates were centrifuged at 12,000 rpm for 30 min at 4°C. Then the protein concentration was measured by using the Bradford assay. An equal amount of protein was loaded on 6%–15% sodium dodecyl sulfate–polyacrylamide gel electrophoresis (SDS-PAGE) gels, transferred to PVDF membranes, and then blocked using 5% skim milk for 1 h. Afterward, the membranes were incubated overnight at 4°C with the following primary antibodies: fatty acid synthase (FAS), SREBP1c, CCAAT/enhancer-binding proteins (C/EBPα), TNF-α (Cell Signaling Technology, Inc., Danvers, MA, United States, 1:500), carnitine palmitoyltransferase I (CPT1), acetyl-CoA carboxylase (ACC), phosphorylated ACC (p-ACC), PPARα (Santa Cruz Biotechnology, Santa Cruz, CA, United States, 1:200), α-tubulin (Sigma Aldrich, St. Louis, MO, United States 1: 4,000), anti-IL-6 (IL-6), and anti-C reactive protein (CRP) (Abcam, Cambridge, United Kingdom). After overnight incubation, the membranes were washed and incubated with the secondary antibody for 1 h at 20–25°C. The protein bands were detected using a ChemiDoc XRS+ imaging system (Bio-Rad, CA, United States) and Syngene GeneSnap (Syngene, Cambridge, United Kingdom).

### Reverse transcription–quantitative polymerase chain reaction (RT-qPCR)

RNA from cells was extracted using the TRIzol^®^ reagent (Invitrogen, CA, United States), following manufacturer instructions. Isolated RNA was used to synthesize cDNA using the PrimeScript™ RT reagent kit (Takara Bio, Otsu, Japan), according to the manufacturer’s protocol. cDNA was converted from 1 µg RNA to a volume of 20 µl. Quantitative polymerase chain reaction (qPCR) experiment was done using SYBR^®^ Premix Ex Taq™ (Takara Bio) on an Mx3005P qPCR system (Agilent Technologies). The thermocycling conditions were as follows: initial denaturation at 95 °C for 30 s, followed by 40 cycles of 95 °C for 15 s and 60°C for 1 min. The primer sequences used for amplification are as follows: SREBP1c forward 5'-GCG​CCT​TGA​CAG​GTG​AAG​TC-3' and reverse 5'-GCC​AGG​GAA​GTC​ACT​GTC​TTG-3'; FAS forward 5'-CCC​CTG​ATG​AAG​AAG​GAT​CA-3' and reverse 5'-GCC​AGG​GAA​GTC​ACT​GTC​TTG-3'; and C/EBP-α forward 5'-TGG​ACA​AGA​ACA​GCA​ACG​AGT​A-3' and reverse 5'-ATT​GTC​ACT​GGT​CAG​CTC​CAG-3'. The mRNA levels were normalized to the values against α-tubulin, which was used as an internal control, and the data were justified by using the 2^−ΔΔCq^ method.

### Animal study design

Several 4-week-old male C57BL/6 mice were purchased from Raon Bio (Gyeonggi-do, South Korea) and were maintained under controlled environmental conditions: temperature of 22 ± 1°C, humidity of 50 ± 5%, and 12-h light/12-h dark cycle. The mice were permitted *ad libitum* access to food and water. After 1 week of acclimation period, mice were randomly divided into three groups: control group (C; n = 6) which was fed a rodent diet (10% kcal fat, Research Diets, New Brunswick, NJ, United States), diabetic group (DMC; n = 6), and treatment group (KHAG; n = 6) which was fed with a high-fat–containing rodent diet (60% kcal fat, Research Diets, New Brunswick, NJ, United States). After 4 weeks of diet treatment, diabetic and treatment groups were twice injected with 60 mg/kg streptozotocin (STZ) into the peritoneum by a 3-week interval in citrate buffer (pH 4.5) to induce T2DM. Simultaneously, the control group was injected with only citric acid buffer. The mice groups are as follows: 1) control groups; 2) diabetic groups, 60% kcal high-fat diet (HFD) + 60 mg/kg STZ; and 3) treatment groups, HFD +60 mg/kg STZ +10 mg/kg KHAG-04. The distilled water or KHAG-04 was orally administrated daily for 12 weeks.

During supplementation, body weight, food intake, and fasting blood glucose levels from the tail vein were monitored once a week. At the end of the treatment of 12 weeks, the animals were anesthetized by inhalation with diethyl ether (Duksan, Seoul, South Korea). The blood samples were collected by using a heparin (Sigma Aldrich, St. Louis, MO, United States)-coated syringe by cardiac puncture and were centrifuged at 850 g at 4°C for 10 min to obtain plasma. For protein extraction, hepatic tissue was frozen in liquid nitrogen and stored at −80°C before the experiment.

### Oral glucose tolerance test (OGTT) and insulin tolerance test (ITT)

The OGTT mice were fasted for 12 h, following which glucose solution was administrated at a 1 g/kg concentration. The blood glucose levels were measured at intervals of 0, 15, 30, 60, 90, and 120 min *via* administration of glucose with a glucometer from the mice’s tail vein. For the ITT, after a 12-h fasting period, mice were intraperitoneal injected with an insulin solution of 1.0 U/kg. Then blood glucose levels were measured as described in the OGTT method.

### Determination of glyoxal (GO) levels in the liver

The glyoxal (GO) amounts were analyzed using high-performance liquid chromatography (HPLC, Waters Corp., MA, United States), according to the protocol by Dhar et al. (2009). The standard of unreacted MGO in the samples was detected by the ratio of the peak area of quinoxaline to that of 5-methylquinoxaline (5-MQ). It is known that o-phenylenediamine (o-PD) reacts with GO to form quinoxaline (doi; 10.5923/j.ajoc.20150501.03). Quinoxaline and 5-MQ were external and internal standards, respectively. The liver tissues were weighed and homogenized and incubated with 0.45M perchloric acid (PCA) and 10 mM o-PD for 24 h at 20–25°C in the dark. Then samples were centrifuged, filtered, and injected into the HPLC system. The samples were analyzed using a YMC-Triart C18 column (250 mm × 4.6 mm, 5 μm; YMC Co. Ltd. Japan) with 20% acetonitrile (ACN) as the mobile phase at a detection wavelength of 315 nm. The column temperature was maintained at 30°C with a flow rate of the mobile phase being 1 ml/min.

### Histological analysis

Animal liver and kidney tissues were fixed with 10% neutral buffered formalin, embedded in paraffin, and sectioned into 5-µm-thick sections. The sections were stained using hematoxylin and eosin (H&E), Masson’s trichrome (MT), and periodic acid–Schiff (PAS). Then the sections were stained using oil red O. The stained tissue slides were examined under a Nikon Eclipse 80i microscope (Nikon, Tokyo, Japan) at ×100 magnification.

### Protein and ligand preparation for docking

Before docking analysis, the crystal 3D structure of human protein SREBP1, FAS, and C/EBPα were retrieved from the Protein Data Bank (PDB ID: 1AM9, 4PIV, and 1NWQ). The structure of proteins rearrangement was obtained by using the Discovery Studio Visualizer. For docking, all water molecules were deleted and polar hydrogen atoms were added to the proteins. The modified proteins were saved in the PDB format. For ligand preparation, we used ChmeDraw software to draw and convert the 2D ligand structure into a 3D form. The 3D structure of the ligand (KHAG-04) was saved in the PDB format.

### Molecular docking

AutoDock (4.2.6) was used to conduct a molecular docking study between ligand and proteins. Initially, protein and ligand were uploaded in AutoDock Vina, following grid maps preparation. AutoGrid supplied with AutoDock Vina was used for grid box preparation at 126, 126, and 126 Å for X, Y, and Z, respectively. The output files after AutoGrid run were saved in the PDBQT format. Then these files were used for the AutoDock run. The Lamarckian genetic algorithm (LGA) was chosen for the best conformers. In the docking process, all other parameters were used as a default setting in AutoDock Vina. Windows 10 operating system was used for AutoDock 4.2.6 and AutoDock Vina. The interactions between protein and ligand were shown using the Discovery Studio Visualizer.

### Statistical analysis

The data are expressed as means ± standard error of the mean (SEM). Statistical comparisons were performed between control and several groups using Bonferroni’s test for multiple comparisons of one-way analysis of variance (ANOVA) using GraphPad Prism 5.0 (GraphPad Software Inc., CA, United States). *p* values < 0.05 were considered statistically significant.

## Results

### Synthesis of KHAG compounds as AGEs breaker

**SCHEME 1:**
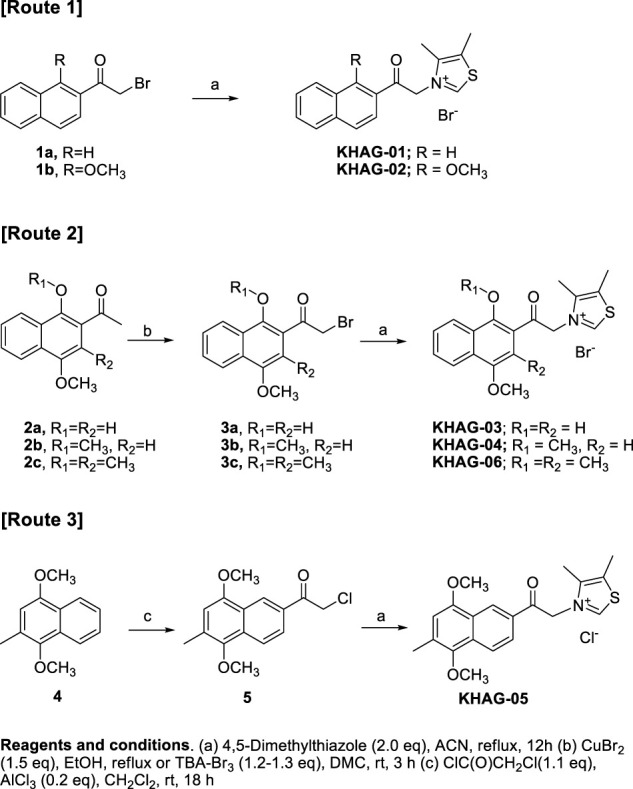
Synthesis scheme for KHAG-01 to KHAG-06.

Novel naphthalene-2-acyl thiazolium derivatives (KHAG series) are designed to make analogs of alagebrium, which has a phenacyl thiazolium structure. A typical synthesis of target molecules (KHAG-01, KHAG-02, KHAG-03, and KHAG-04) includes the acylation of substituted naphthalene (Murakami et al., 1988; Boyer et al., 2000; Qandil and Fakhouri, 2012), α-bromination of 2-acylnaphthalene intermediate (Ran et al., 2016), and coupling reaction with 4,5-dimethylthiazole, as shown in Scheme 1.

On the other hand, Friedel-Crafts acylation reaction of compound 4 was attempted to synthesize 1,4-dimethoxy-3-methylnaphthalene-2-acyl compound KHAG-06, but intermediate 2c acylated at position 2 was not produced. Instead of the acyl group substitution at position 2, Friedel-Crafts acylation at position 7 was observed. Hence, we performed Friedel-Crafts acylation of 4 with chloroacetyl chloride to give an intermediate 5 and then coupled it with 4,5-dimethylthiazole to afford thiazolium salt KHAG-05. Thus, a thiazolium compound KHAG-06 containing the methyl group substituted at the C-3 position of the naphthalene ring was prepared from the starting compound 2c, which was prepared from 2-bromo-1,4-dimethoxy-2-methylnaphthalene (Jung et al., 2005; Kim et al., 2020). The α-bromination of 2c gave intermediate 3c, followed by a coupling reaction with 4,5-dimethylthiazole to provide the thiazolium salt KHAG-06.

**SCHEME 2:**
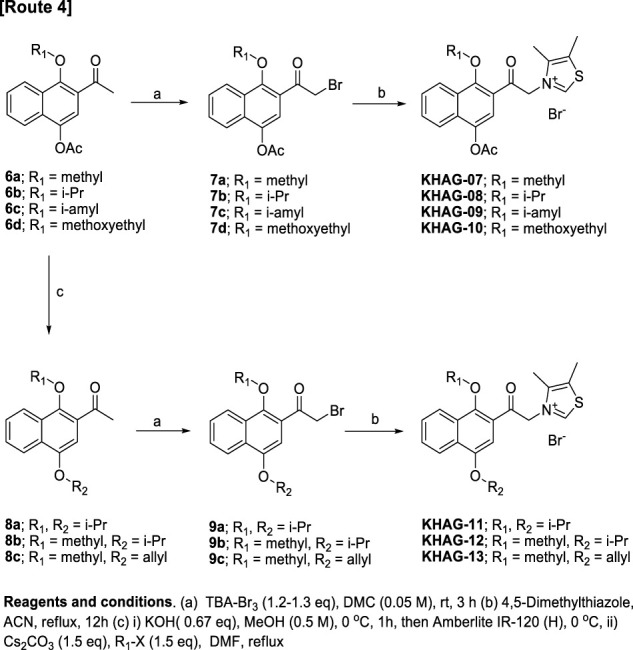
Synthesis scheme for KHAG-07 to KHAG-13.

4-Acetoxy-1-alkyoxy- or 1,4-dialkoxy-2-acylnaphthalen thiazolium salts, KHAG-07 to KHAG-13, were prepared in the same manner described in Scheme 1, except for o-alkylation methods (Scheme 2). In this synthesis route 4, a starting compound 6 was prepared by Fries rearrangement of 1,4-diacetoxy naphthalene, followed by o-alkylation (Paduraru et al., 2008). The bromination of 6a–d by TBA-Br_3_ gave acylbromide intermediate 7a–d, and the following coupling reaction of 7a–d with 4,5-dimehtylthiazole provided KHAG-07, -08, -09, and -10, respectively. In addition, 1,4-dialkoxynaphthalene derivatives having different dialkoxy groups at positions 1 and 4 were also prepared from intermediate 6a–c. Hydrolysis of compounds 6a–c produced 4-monoalkoxy intermediate 7a–c, and the o-alkylation of intermediate 7a–c followed by α-bromination of the corresponding intermediate 8a–c gave the acylbromide intermediate 9a–c. Finally, coupling of intermediate 9a–c with 4,5-dimethylthiazole afforded KHAG-11, -12, and -13, respectively.

### Effect of KHAG-04 on the breakdown of MGO and GO-AGEs

**FIGURE 2:**
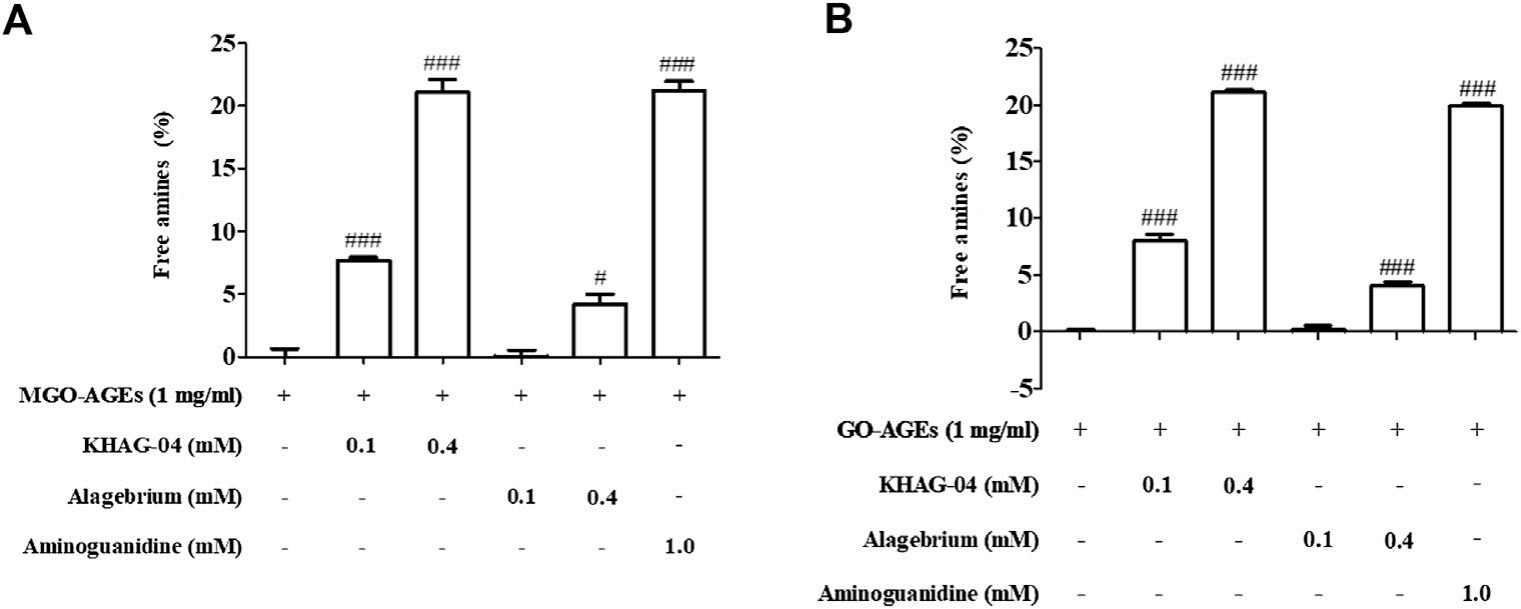
Effects of KHAG-04 in methylglyoxal (MGO)- and glyoxal (GO)-induced advanced glycation end products (AGEs). **(A,B)** Effects of KHAG-04 on the *in vitro* breakdown of MGO- and GO-AGEs were measured using an AGEs breaker assay; graph is presented as an increase of free amines in KHAG-04 treatment sample as compared to GO and MGO-AGEs samples. All data are indicated as mean ± SEM (^#^ *p* < 0.05 and ^###^ *p* < 0.001 vs GO and MGO-AGEs).

MGO/GO-AGEs breakdown potential of synthetic KHAG compounds was evaluated by measuring the level of free amines released following degradation of respective AGEs. The 13 synthetic KHAG compounds underwent screening, and among them, KHAG-04, which has a 1,4-dimethoxynaphthalene moiety, showed the highest free amines released percentage (Supplementary Table S1,2) against both of the AGEs types; hence, we selected this novel synthetic compound KHAG-04 for further experiments. As shown in Figure 2, MGO-AGEs or GO-AGEs (1 mg/ml) solution was reacted with several concentrations (0.1 and 0.4 mM) of KHAG-04, alagebrium, and aminoguanidine (1.0 mM) for 24 h at 37°C. KHAG-04 treatment significantly increased the breakdown of MGO and GO-AGEs in a dose-dependent manner (Figure 2). The AGE breakdown potential of KHAG-04 is almost 4- to 5-folds higher in both the treated concentration (0.1 and 0.4 nM) in relation to alagebrium. Similarly, the potential of 0.4 mM of KHAG-04 is higher or almost similar to that of 1 mM of aminoguanidine (positive control), suggesting that it has more than 2-fold higher potential than that of the commercial positive control. Altogether, the MGO/GO-AGEs breakdown potential of KHAG-04 is almost 4-fold higher than that of alagebrium (based on free amine released) and 2-fold higher than that of aminoguanidine (based on concentration).

### Effect of KHAG-04 on nitrite production and pro-inflammatory cytokines in LPS- and AGE-stimulated Raw 264.7 cells

**FIGURE 3:**
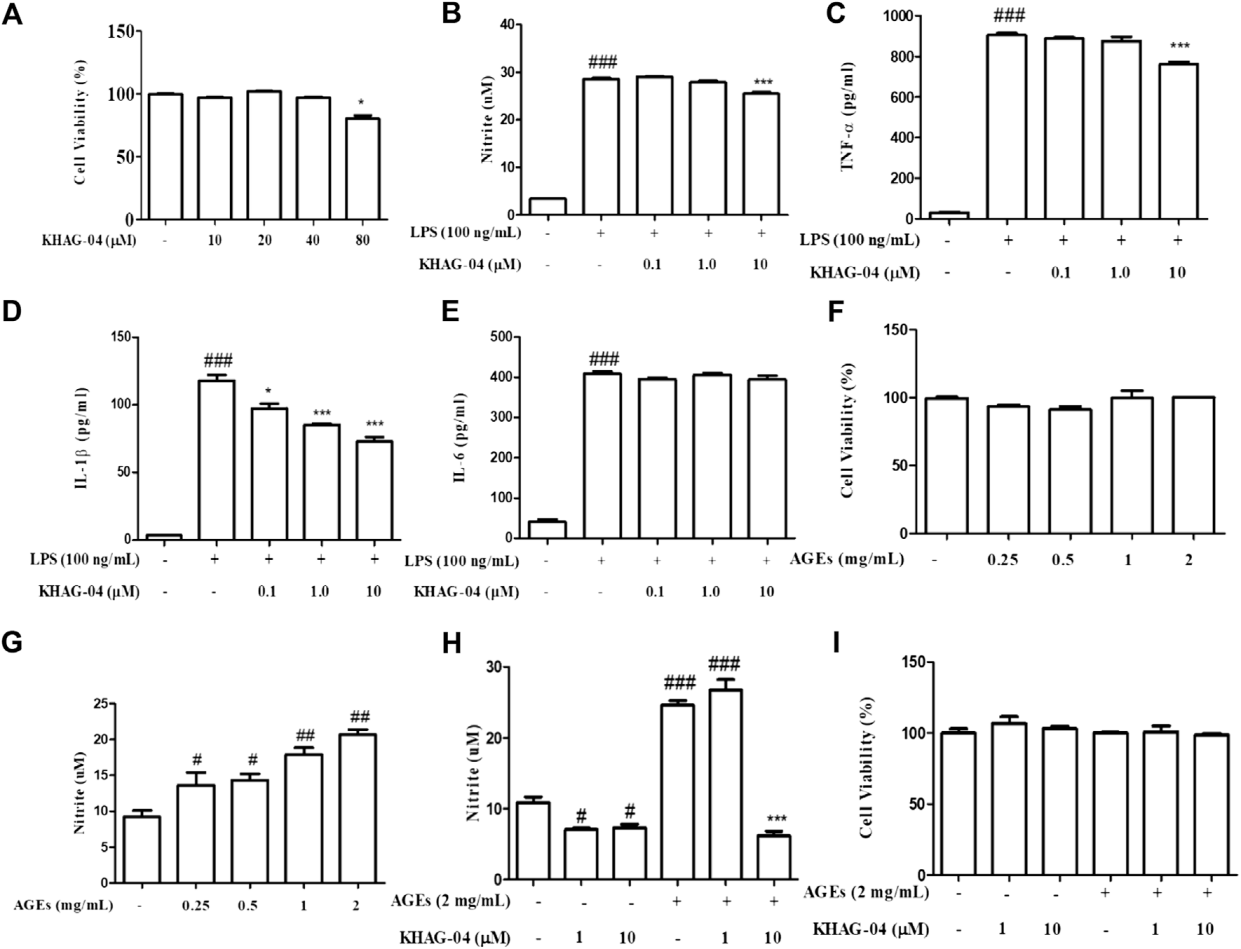
Effects of KHAG-04 on LPS- and AGE-induced nitrite production and pro-inflammatory cytokines secretion in Raw 264.7 cells. Raw 264.7 cells were pre-treated with 0.1, 1, and 10 μM of KHAG-04 for 1 h and stimulated with 100 ng/ml of LPS for an additional 23 h. **(A)** Cell viability in Raw 264.7 cells was evaluated using the MTT assay. **(B)** Nitrite production was measured by using the nitrite assay. **(C–E)** The secretion of cytokines IL-1β, IL-6, and TNF-α in the supernatant was measured according to the manufacturer’s ELISA assay kit protocol. **(F)** RAW 264.7 cells were stimulated with different concentrations of MGO-AGEs (0.25, 0.5, 1, and 2 mg/ml) for 24 h. **(G)** Nitrite production at different concentrations of AGEs was measured by using the nitrite assay. **(H)** Cells were pre-treated with KHAG-04 (1 and 10 uM) for 1 h and stimulated at indicated concentrations (2 mg/ml) of MGO-AGEs for an additional 23 h for nitrite measurement **(I)** Cell viability was evaluated in presence of MGO-AGEs and KHAG-04. All data are indicated as mean ± SEM (#, ##, *p* < 0.05 and ^###^ *p* < 0.001 vs Control; *, **, ^**^ *p* < 0.05 and ^***^ *p* < 0.001 vs 100 ng/ml LPS and 0.5 mg/ml AGES).

The anti-inflammatory and cytotoxic effect of KHAG-04 was evaluated against LPS-activated macrophages by measuring the level of inflammatory biomarkers including nitrite (NO) production, TNF-α, IL-1β, IL-6, and cell viability. KHAG-04 did not show any cellular toxicity in the normal condition until 40 µM as well as LPS-activated microglia (Figure 3A). We used the three lowest concentrations of KHAG-04 to check its anti-inflammatory effects in RAW 264.7 cells. Treatment of KHAG-04 significantly lowered the NO production in the LPS-activated Raw 264.7 cells without cellular toxicity (Figure 3B) at 10 µM. LPS treatment dramatically induced the production of inflammatory cytokines including TNF-α, IL-1β, and IL-6 (Figures 3C–E). KHAG-04 treatment significantly lowered the production of TNF-α and IL-1β with high potency but did not alter the level of IL-6 against LPS-activated macrophages. All the treated concentrations even 0.1–10 μM of KHAG-04 showed a significant potency to lower the IL-1β production. In addition, we cheeked KHAG-05 effects on the same condition in Raw 264.7 cells. Though KHAG-05 showed inhibitory effects on nitrite production and inflammatory cytokines IL-1β, the results were not that promising as compared to KHAG-04 (Supplementary Figure S1). We also examined the cell toxicity and nitrite (NO) production in the presence or absence of MGO-AGEs in RAW 264.7 cells. MGO-AGEs showed no toxic effects on RAW 264.7 cells in the presence or absence of KHAG-O4 in a normal and activated state (Figure 3F). MGO-AGEs strongly induced NO production in a dose-dependent manner in RAW 264.7 cells (Figure 3G). KHAG-04 treatment remarkably reduced NO production in RAW 264.7 cells at 10 μM, while it has no damaging effects on cell viability (Figures 3H,I).

### Effect of KHAG-04 on FFA-induced lipogenesis in HepG2 cells

**FIGURE 4:**
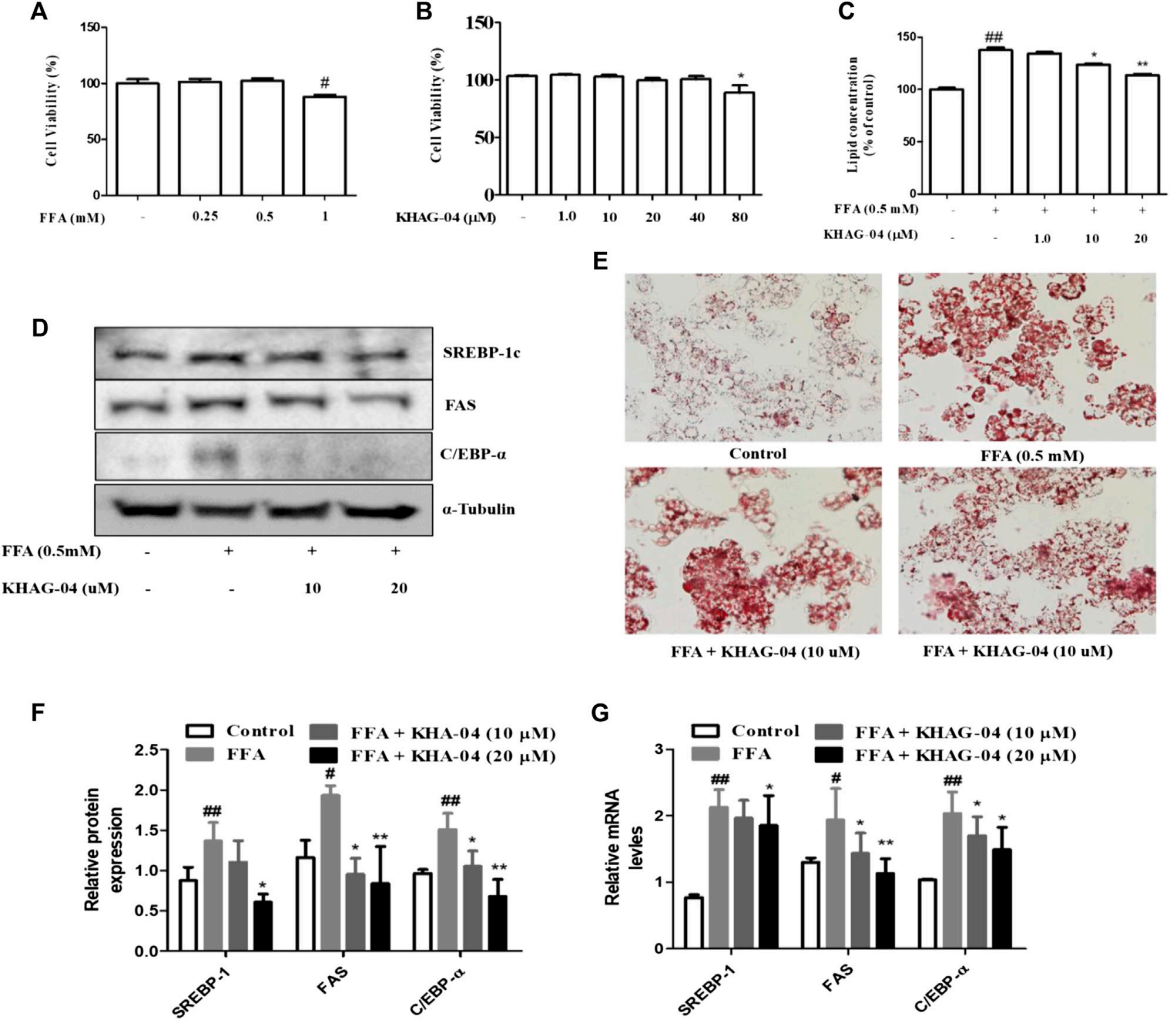
Effects of KHAG-04 on FFA-induced lipid metabolism–related markers in HepG2 cells. **(A,B)** Cell viability of FFA and KHAG-4 in Hep-G2 cells was evaluated using the MTT assay. **(C,E)** HepG2 cells were pretreated with 10 and 20 μM of KHAG-04 for 1 h and then stimulated with FFA 0.5 mM for 23 h. FFA-induced lipid droplet accumulation was observed using oil red o staining (×100 magnification). Intracellular lipid concentrations were quantified by the spectrometer at 540 nm **(C)** KHAG-04 inhibited FFA-induced protein expression of SREBP-1c, FAS, and C/EBP-α. A Western blot analysis was performed using the indicated antibodies. The mRNA expression of prime metabolic genes (SREBP-1c, FAS, and C/EBP-α) was detected after 24 h. Actin was used as a housekeeping gene. All data are indicated as mean ± SEM (^##^ *p* < 0.001 vs Control; ^**^ *p* < 0.001 vs 0.5 mM FFA).

We investigated the effects of KHAG-04 against FFA-induced lipid droplets accumulation, its related factors proteins, and mRNA expression in HepG2 cells. FFA showed toxicity at 1 mM in Hep-G2 cells; we selected 0.5 mM of FFA for further study (Figure 4A). KHAG-04 did not show any toxicity until 80 µM (Figure 4B). Oil red O staining, for measuring lipid accumulation, indicated the FFA treatment markedly induced lipid droplets accumulation in HepG2 cells. KHAG-04 pre-treatment drastically reduced FFA-induced lipid accumulation only at 10 and 20 µM but lipid droplets were not reduced at 1 µM in HepG2 cells (Figures 4C,E). Moreover, KHAG-04 pre-treatment significantly reduced FFA-induced lipogenesis transcription factors (SREBP-1_C_) and proteins’ (FAS and C/EBP-α) expression levels in Hep-G2 cells (Figures 4D,F). In addition, KHAG-04 seems to reduce FFA-induced mRNA expression levels of SREBP-1C, FAS, and C/EBP-α in Hep-G2 cells (Figure 4G).

### Effect of KHAG-04 on MGO-AGE–induced lipogenesis in HepG2 cells

**FIGURE 5:**
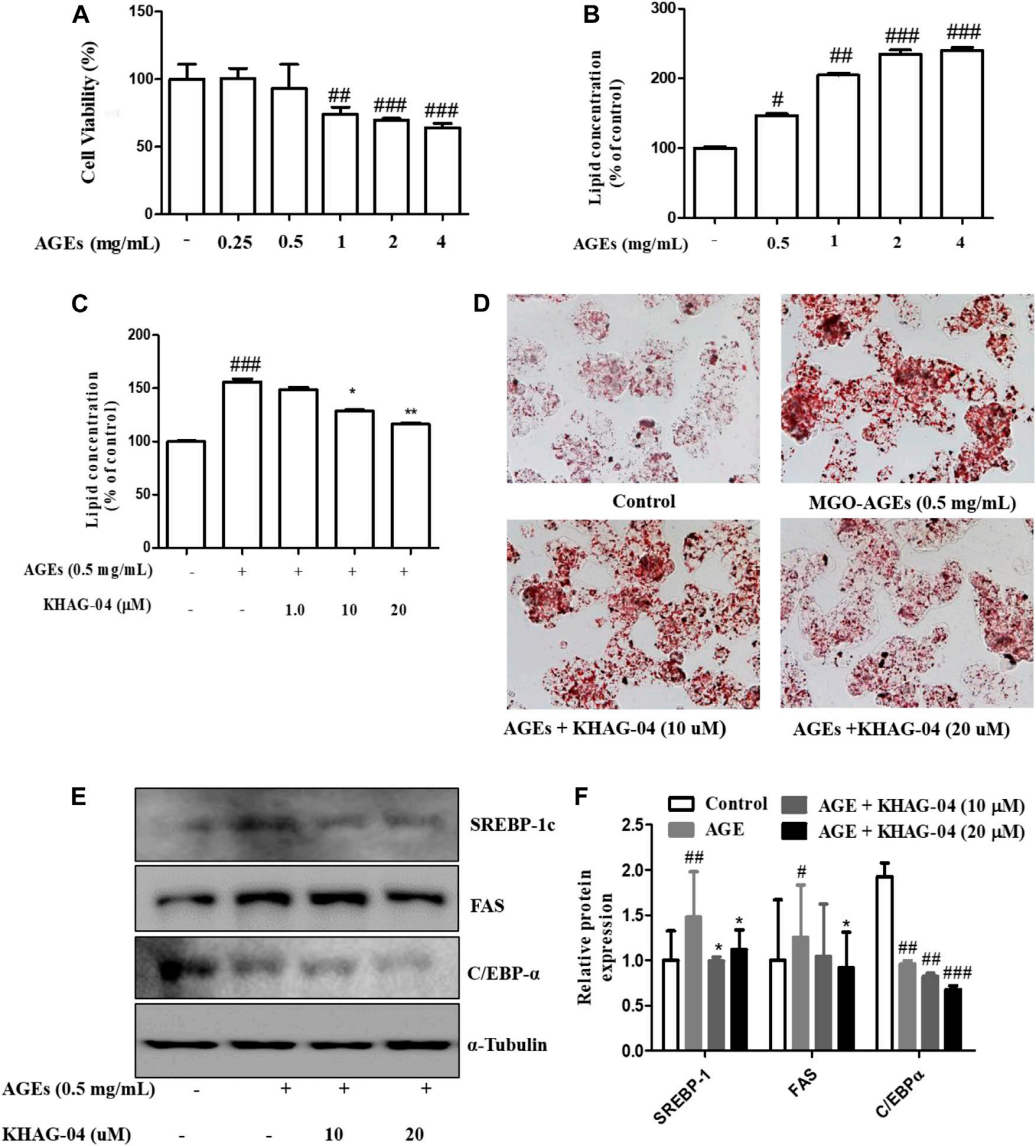
Effects of KHAG-04 on AGE-induced metabolism–related markers in HepG2 cells. **(A)** Cell viability of AGEs in Hep-G2 cells was evaluated using the MTT assay. **(B)** Intracellular lipid concentrations in presence of a different concentration of AGEs were quantified by the spectrometer at 540 nm. **(C,D)** HepG2 cells were pretreated with 10 and 20 μM of KHAG-04 for 1 h and then stimulated with AGEs (0.5 mg/ml) for 23 h. AGE-induced lipid droplet accumulation was observed using oil red o staining (×100 magnification). Intracellular lipid concentrations were quantified by the spectrometer at 540 nm **(E)** KHAG-04 inhibited AGE-induced protein expression of SREBP-1c, FAS, and C/EBP-α. A Western blot analysis was performed using the indicated antibodies. **(F)** Densitometry graph of figure **(E)**. All data are indicated as mean ± SEM (^#^ *p* < 0.005 vs Control, ^###^ *p* < 0.001 vs Control, and ^*^ *p* < 0.05 and ^**^ *p* < 0.001 vs 0.5 mg/ml AGEs).

Next, we were curious to see whether MGO-AGEs (AGEs) can induce any lipogenic effects in Hep-G2 cells and if KHAG-04 has any protective effects on that. First, cell toxicity of AGEs at different concentrations (0.25, 0.5, 1, 2, and 4 mg/ml) in Hep-G2 cells was assayed, and the cell death started at 1 mg/ml (Figure 5A). The lipid concentration was examined with similar concentrations of AGEs. AGEs significantly induced lipid accumulation in Hep-G2 cells in a dose-dependent manner (Figure 5B). From the aforementioned findings, we decided to use 0.5 mg/ml of AGEs for the next experiment. We investigated the effects of KHAG-04 against AGE-induced lipid droplets accumulation and its related factors proteins expression in HepG2 cells. KHAG-04 pre-treatment markedly ameliorates AGE-induced lipid accumulation only at 10 and 20 µM, but lipid droplets were not reduced at 1 µM in HepG2 cells (Figures 5C,D). In addition, KHAG-04 pre-treatment significantly attenuates AGE-induced lipogenesis transcription factors (SREBP-1_C_) and proteins (FAS and C/EBP-α) expression levels in Hep-G2 cells (Figures 5E,F).

### Effect of KHAG-04 on body weight, liver weight, food intake, and fasting blood glucose (FBG) in T2DM mice

The body weight of T2DM groups (before treatment) was significantly increased compared with the control group (CON), whereas fasting blood glucose and food intake were not significantly increased. Body weight, liver weight, and food intake of the KHAG-04 treated group were noticed to be decreased in comparison to the T2DM/DMC treated group but it was not significant (Table 1).

**TABLE 1:** Effect of KHAG-04 on body weight and food intake in the type 2 diabetes (T2DM) mice model.

Group	CON	DMC	KHAG-04
Body weight (g)			
Before treatment	28.32 ± 1.05	35.3 ± 4.23^##^	33.96 ± 1.66^#^
After treatment	30.43 ± 2.50	40.70 ± 6.39^#^	38.19 ± 4.77
Gain	2.11 ± 1.92	5.34 ± 2.55	4.23 ± 3.35
Liver weight (g)	0.96 ± 0.13	1.48 ± 0.43^#^	1.24 ± 0.18
Food intake (g/day)	2.99 ± 0.08	2.73 ± 0.10	2.36 ± 0.27
Fasting blood glucose (FBG) (mg/dl)	161.80 ± 28.45	220.20 ± 55.67	235.20 ± 39.51

The data of each experiment is presented as the mean ± SEM of three independent experiments (^#^ *p* < 0.05, ^##^ *p* < 0.01 vs control). CON, control mice; DMC, dichloromethane; type 2 diabetes mice (KHAG-04), type 2 diabetes mice supplemented with a 10 mg/kg/day of KHAG-04.

### Effect of KHAG-04 on glucose homeostasis and insulin sensitivity in T2DM mice

**FIGURE 6:**
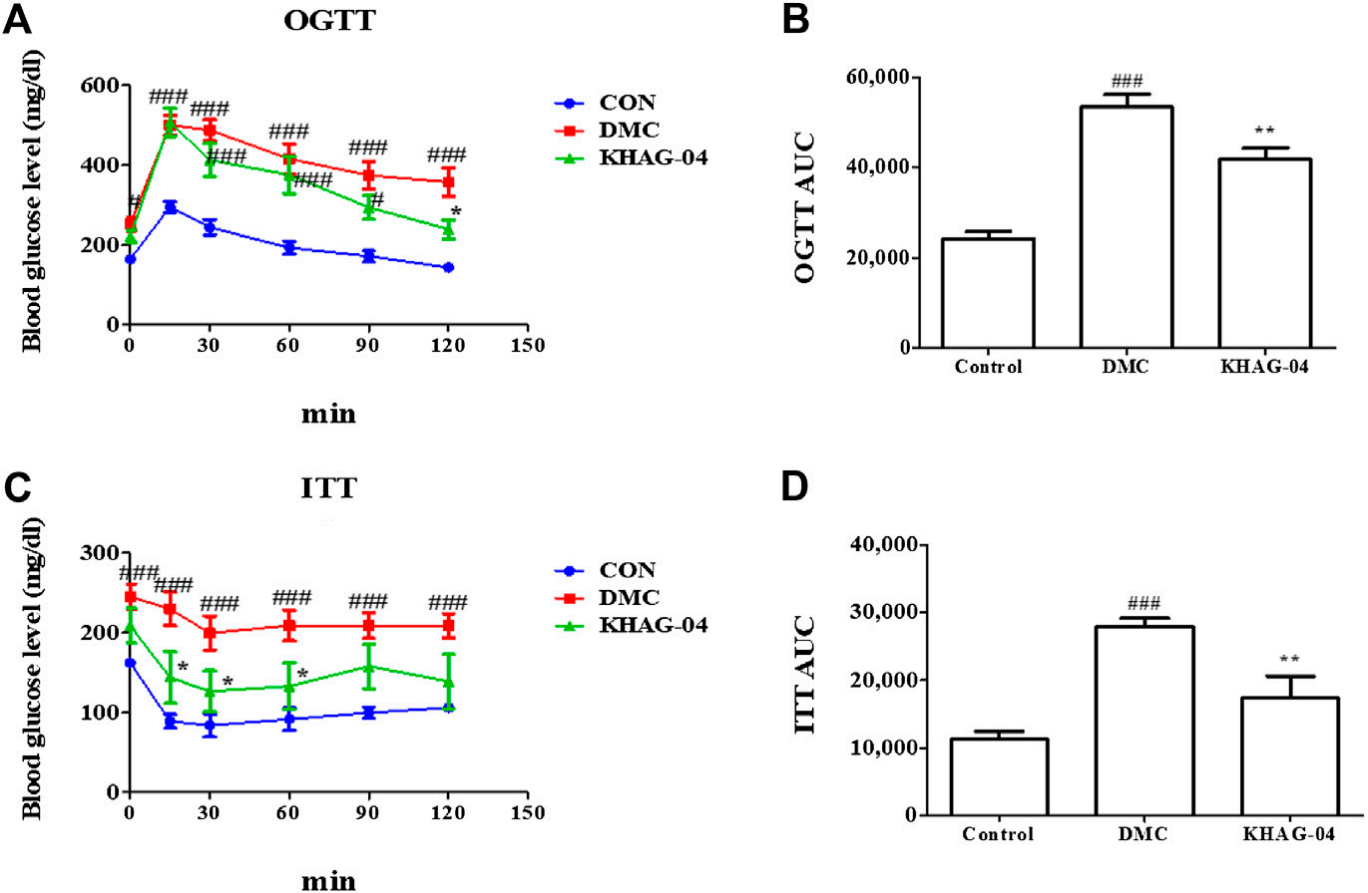
Effects of KHAG-04 on the oral glucose tolerance test (OGTT) and the insulin tolerance test (ITT) in T2DM mice. 60% kcal high-fat diet (HFD) and 60 mg/kg streptozotocin (STZ)-stimulated T2DM mice were treated with vehicle and KHAG-04 (10 mg/kg) for 12 weeks. **(A)** Intraperitoneal insulin tolerance test (ITT), and **(B)** the area under the curve (AUC) of ITT. **(C)** Oral glucose tolerance test (OGTT) and **(D)** the area under the curve (AUC) of OGTT. All data are indicated as mean ± SEM (^#^ *p* < 0.05, ^###^ *p* < 0.001 vs Control; ^*^ *p* < 0.05, ^**^ *p* < 0.01 vs 60% kcal high-fat diet + 60 mg/kg streptozotocin; DMC).

OGTT and ITT were performed on HFD + STZ-induced T2DM mice. As shown in Figure 5, both DMC and KHAG showed higher glucose levels compared to the CON group, where the level of blood glucose level in both OGTT and ITT in the KHAG treated group was significantly lower than that of the DMC group at 120 min (Figures 6A,B). Similarly, the OGTT and ITT AUC of the DMC group were almost 2- to 3-fold higher than the CON group but it was significantly lower in the T2DM animals treated with KHAG-04 (Figures 6C,D) suggesting that KHAG-04 can take part in controlling the glucose homeostasis and insulin sensitivity of the T2DM animals in a positive way.

### Effect of KHAG-04 on glucotoxicity and lipotoxicity related to GO levels in the liver of T2DM mice

To analyze the KHAG-04 with the regulating abilities against HFD + STZ-induced T2DM mice, we measured the level of GO accumulation in the liver of the T2DM mice via HPLC analysis. The level of GO in the liver of the DMC group was significantly increased compared with the CON group (Supplementary Figure S2), but the KHAG group was significantly lower than the DMC group (Table 2). These data showed that KHAG treatment might regulate intracellular GO levels and hence less amount of GO-AGEs can form, and higher GO-AGEs can breakdown, and all these steps ultimately help to ameliorate GO and GO-AGE–related toxicities and secondary damage in T2DM animals.

**TABLE 2:** GO level in the T2DM mice liver.

Sample	Concentration (nmol/mg protein)
CON	33.97 ± 2.32
DMC	47.90 ± 6.96^#^
KHAG-04	35.05 ± 3.96^*^

The data of each experiment is presented as the mean ± SEM of three independent experiments (^#^ *p* < 0.05 vs control, ^*^ *p* < 0.05 vs DMC). CON, control mice; DMC, dichloromethane; type 2 diabetes mice (KHAG-04), type 2 diabetes mice supplemented with a 10 mg/kg/day of KHAG-04.

### Effect of KHAG-04 on hepatic steatosis related to lipid droplet accumulation and collagen deposition in the liver of HFD + STZ-stimulated T2DM mice

**FIGURE 7:**
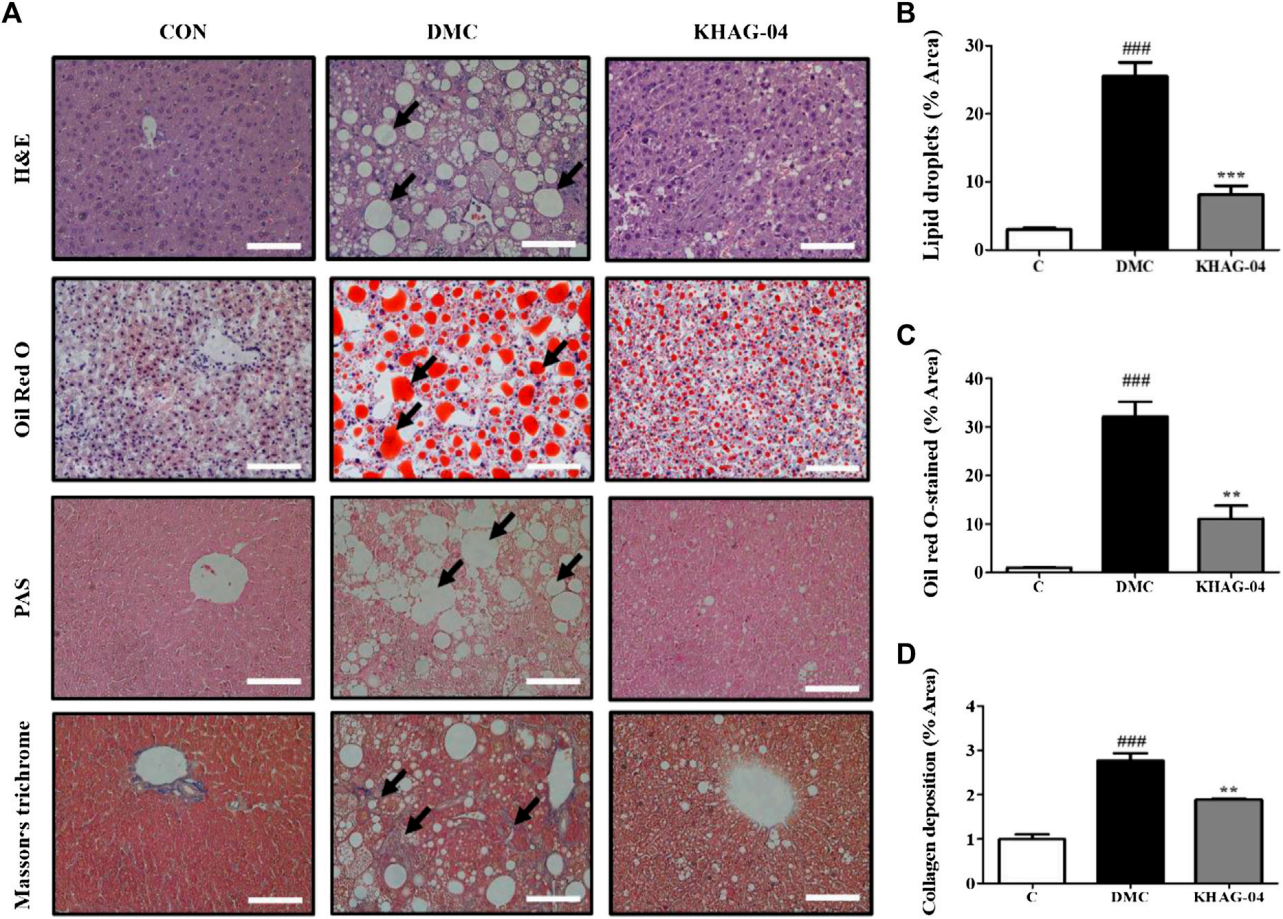
Effects of KHAG-04 on hepatic steatosis in the liver of HFD- and STZ-induced T2DM mice. **(A)** Histological staining of liver sections was performed. Representative H&E, oil red O, PAS, and Masson’s trichrome (MT) stained images are shown. Black arrows show lipid droplets and collagen deposition (×100 magnification). The scale bar indicates 100 μm. Quantitative measurements of lipid droplets **(B)**, oil red O stained **(C)**, and collagen deposition **(D)** values were calculated using ImageJ software. All data are indicated as mean ± SEM (^###^ *p* < 0.001 vs Control; ^**^ *p* < 0.01, ^***^ *p* < 0.001 vs 60% kcal high-fat diet + 60 mg/kg streptozotocin; DMC).

To determine the effect of KHAG-04 against lipotoxicity in T2DM, we performed the H&E, oil red O, PAS, and MT staining in the liver tissue. The lipid droplet accumulation in the liver sections of the DMC group was remarkably increased compared with the CON group. Hepatic lipid deposition generated severe liver injury and it causes liver dysfunction by disturbing the lipid transport, uptake, synthesis, and ballooning function (Yan et al., 2015). These changes were also observed in our study. After treatment of animals with KHAG-04 for 12 weeks, liver histological damages were ameliorated, which was established via the significant improvement of steatosis and ballooning degeneration (Figures 7A–C). Furthermore, the collagen deposition of the liver tissues was checked by MT staining. However, the KHAG group significantly decreased collagen deposition compared with the DMC group (Figures 7A,D). Altogether, results revealed that KHAG-04 treatment can play an important role in ameliorating T2DM-mediated liver toxicity.

### Effect of KHAG-04 on hepatic protein expressions of lipid metabolism related markers in T2DM mice

**FIGURE 8:**
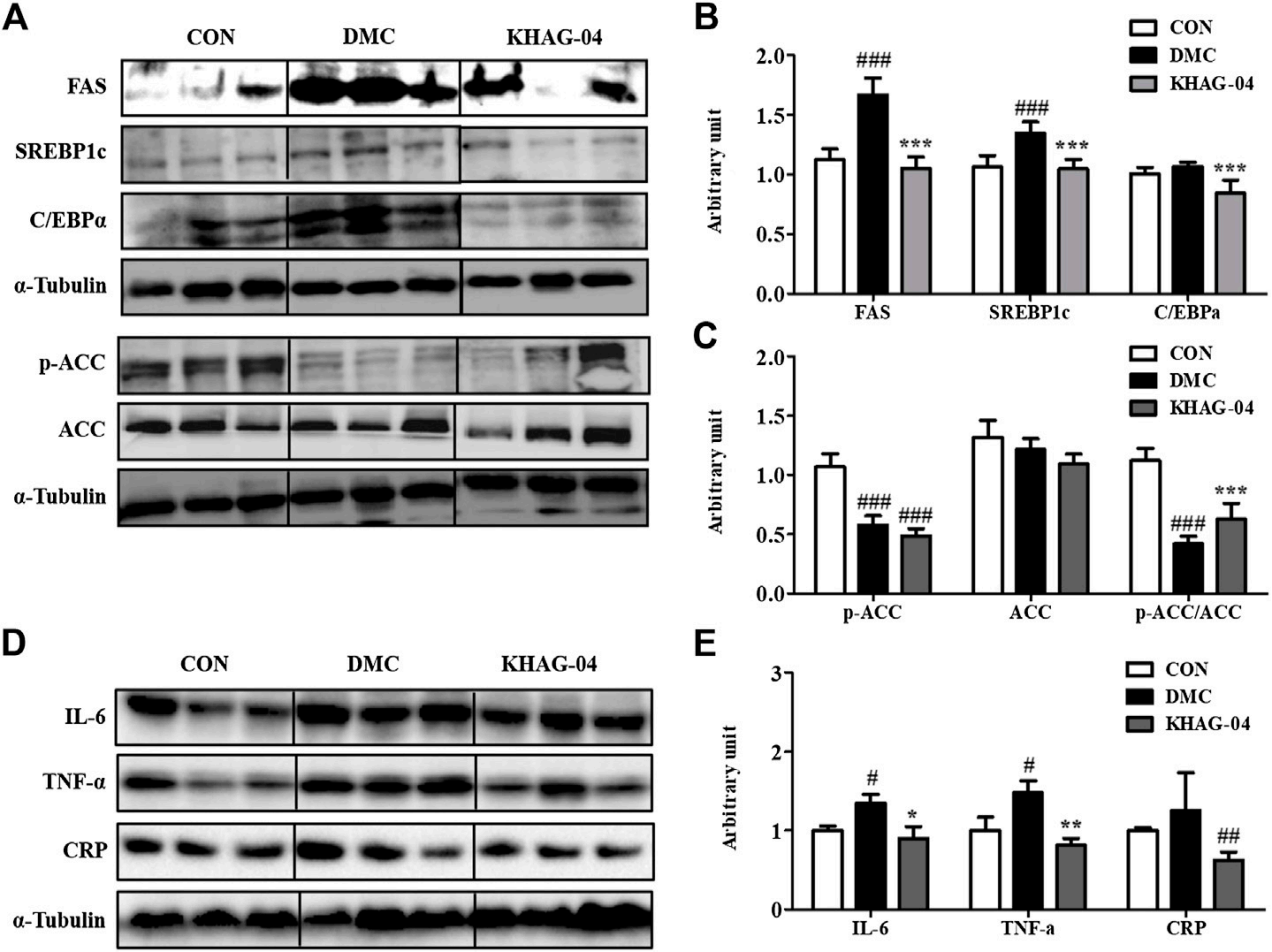
Effects of KHAG-04 on hepatic protein levels of lipid metabolism–related and inflammation-related markers. **(A)** Protein expression of fatty acid synthase (FAS), sterol regulatory element–binding protein1 (SREBP1c), CAAT box/enhancer–binding protein alpha (C/EBPα), and phospho acetyl-CoA carboxylase (p/T-ACC) in liver tissue of T2DM mice. The hepatic protein was measured by Western blot. The bands show the intensity of the bands that were densitometrically measured and normalized to the band levels of α-tubulin **(B,C)**. **(D)** Representative band images indicate the protein expression of NAFLD and pro-inflammatory cytokines–related antibodies (CRP, IL6, and TNF-α) as measured by Western blotting. **(E)** Relative band intensities of CRP, IL-6, and TNF-α. All data are indicated as mean ± SEM (^#^ *p* < 0.05, ^##^ *p* < 0.01, ###*p* < 0.001 vs Control; ^*^ *p* < 0.05, ^**^ *p* < 0.01, ***p < 0.001 vs 60% kcal high-fat diet + 60 mg/kg streptozotocin; DMC).

The protein expression levels of FAS, SREBP1c, and C/EBPα in the DMC group were significantly higher than those in the NC group. KHAG-04 treatment significantly lowered them compared to the DMC group at 10 mg/kg (Figures 8A,B). The protein levels of p-ACC and ACC were decreased, and the ratio of p-ACC/ACC was decreased in the DMC group compared to those of the NC group, but it was not normalized compared to the DMC group (Figures 8A,B).

### Effect of KHAG-04 on liver injury and pro-inflammatory cytokines protein expression in T2DM mice

To investigate the effects of KHAG-04 on NAFLD and pro-inflammatory cytokines in T2DM mice, we evaluated the protein levels of CRP, IL-6, and TNF-α in the experimented animal tissues. CRP is the biomarker of liver injury. The CRP protein expressions were increased compared to the CON group though it was not significant enough. The KHAG group showed a reduced level of CRP in comparison to the DMC group alone (Figures 8D,E). In the case of pro-inflammatory cytokines, the DMC group significantly increased the protein expression levels of IL-6 and TNF-α, but the protein expression of these inflammatory cytokines was significantly lower in the T2DM animal treated with KHAG-04 (Figures 8D,E). Altogether, these results suggested that KHAG-04 can lower the conditions of NAFLD and inflammation in the liver by reducing the level of NAFLD and pro-inflammatory biomarkers such as CRP, IL-6, and TNF-α.

### KHAG-04 and lipid metabolism–related proteins interactions via molecular docking studies

**FIGURE 9:**
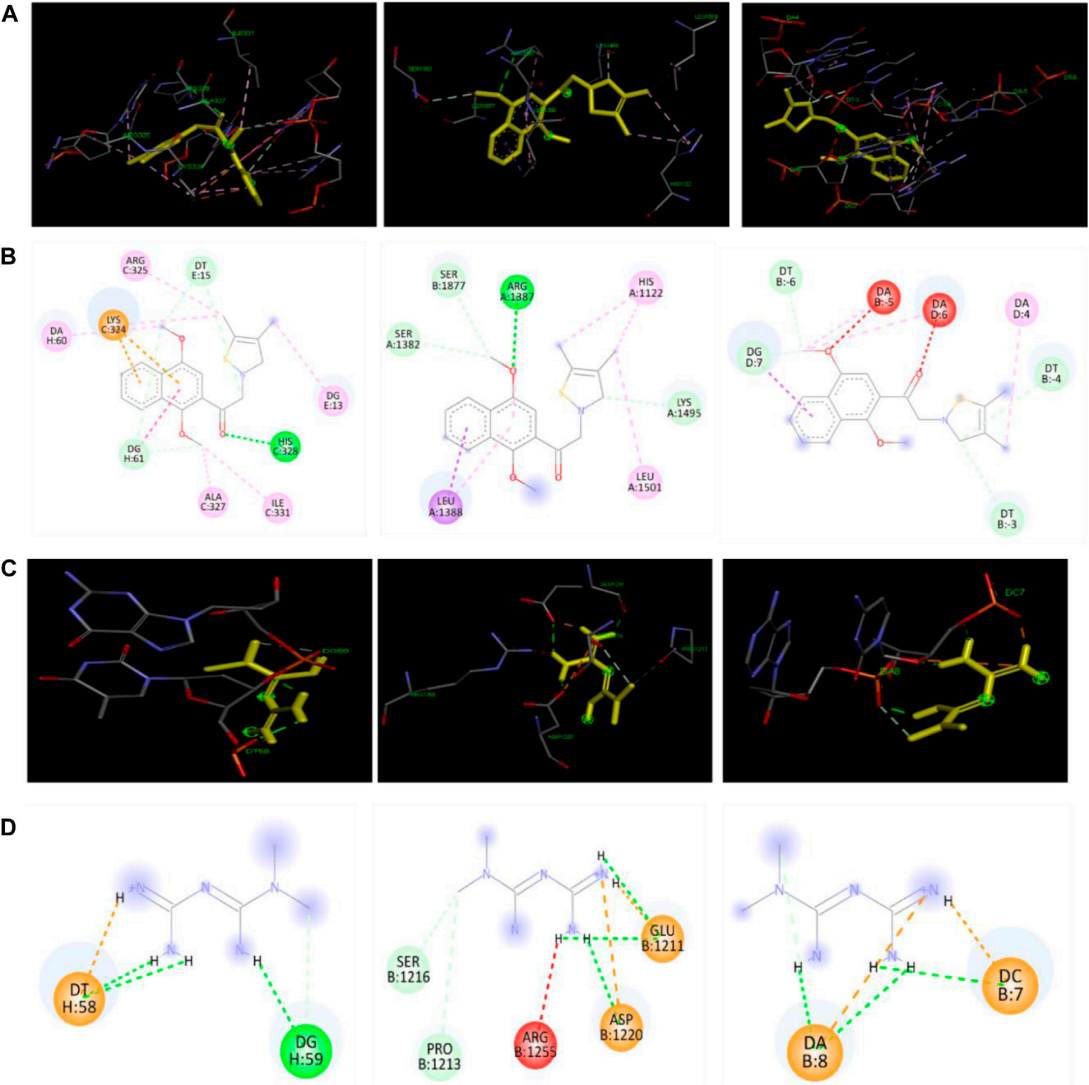
Docking interactions of KAHG-04 and metformin with SREBP1C, FAS, and C/EBPα. **(A)** 3D interaction of KHAG-04 (dark Yellow) with SREBP1C (left panel), FAS (middle panel), and C/EBPα (right panel). **(B)** 2D structural presentation of the amino acid residues and nucleotides interaction of KHAG-04 with SREBP1C (left panel), FAS (middle panel), and C/EBPα (right panel). **(C)** 3D structural interaction of metformin with SREBP1C (left panel), FAS (middle panel), and C/EBPα (right panel). **(D)** 2D structural presentation of the amino acid residues and nucleotides interaction of metformin (dark Yellow) with SREBP1C (left panel), FAS (middle panel), and C/EBPα (right panel). Bold green dot lines represent a conventional hydrogen bond, light green dot lines represent a carbon–hydrogen bond, pink dot lines represent a Pi-Pi sigma bond, light pink dot lines represent a Pi-alkyl bond, brown dot lines represent a Pi-cation bond, red dot lines represent an unfavorable acceptor-acceptor bond, and light yellow dot lines represent a salt bridge bond. All data shown here are from three independent experiments.

The best docking site or mode of KHAG-04 and metformin with SREBP1_C_, FAS, and C/EBPα is displayed in Figure 9. The docking analysis allowed us to identify several types of interactions between ligand and protein such as hydrogen bonding, Pi-Pi T-shaped bonding, Pi-sigma bonding, Pi-Alkyl bonding, salt bridge, and hydrophobic interactions. KHAG-04 interacted with amino residue HIS328, ARG325, ALA327, ILE 331, LYS324, and nucleotide DA60, DG13, DT15, DG61 of SREBP1_C_, where it formed one hydrogen with HIS328, as it is highly conserved among helix-loop-helix protein, releasing the binding energy of −7.84 Kcal/Mol. Likewise, KHAG-04 interacted with amino residue ARG1387, SER1877, SER1382, LYS149, LEU 1388, LEU 1501 of FAS, where it formed one hydrogen with ARG1387, releasing the binding energy of −7.20 Kcal/Mol. Moreover, KHAG-04 only interacted with nucleotide DA4, DA5, DA6, DT3, DT4, DT6, and DG7 of C/EBPα releasing the binding energy of −7.32 Kcal/Mol. However, metformin formed two hydrogen bonds with nucleotides DG59 and DT58 of SREBP1_C_, releasing the binding energy of −6.21 Kcal/Mol. Further analysis revealed that metformin interacted with amino residue ARG1255, ASP 1220, GLU 1211, PRO1213, and SER1216 of FAS, and formed two hydrogen bonds with ASP 1220, and GLU 1211. Last, metformin formed two hydrogen bonds with nucleotides DA8 and DC7 of C/EBPα. Metformin binding energy to FAS and C/EBPα was −5.17 Kcal/Mol and −6.10 Kcal/Mol, respectively. These results suggest that KHAG-04 has the lowest binding energy to lipogenic markers as compared to our standard compound metformin.

## Discussion

Metabolic syndrome–like nonalcoholic fatty liver disease (NAFLD) is highly prevalent in T2DM (Dharmalingam et al., 2018). As T2DM and NAFLD exist together like two sides of a coin, hyperglycemia or glucose accumulation is the major reason for the pathogenesis during these conditions. Hyperglycemia being a major pathogenic factor behind both T2DM and NAFLD, the exact relationship and the treatment strategies against NAFLD targeting hyperglycemia has not been well studied. Considerable evidence suggested that hyperglycemia in kiT2DM can induce the level of glucose, triglycerides, and LDL-C which are responsible for the induction of NAFLD (Shams et al., 2011). Hyperglycemia-mediated glucose breakdown increases the production and accumulation of reactive di-carbonyl compounds such as MGO and GO. MGO and GO can interact with amino acids, lipids, nucleic acids, and proteins via glycation or autooxidation in the body and subsequently accelerate the formation and accumulation of AGEs (Singh et al., 2014; Wetzels ert al., 2017). These AGEs mediated toxicities have been reported in NAFLD as well as in T2DM (Kimura et al., 2010). Kimura et al. reported that increased AGEs level in NASH was a clinically relevant biomarker of NAFLD in T2DM patients. Atorvastatin treatment reduced AGEs levels, suggesting AGEs as a biomarker of NASH and it can be targeted for the treatment of NASH, NAFLD as well as complications in T2DM (Kimura et al., 2010). Another independent study also revealed that atorvastatin-mediated inhibition of AGEs levels in NASH with lipidemia helped the efficacy of atorvastatin in the treatment of these conditions (Hyogo et al., 2008). In our study, we also observed that hyperglycemia-mediated AGEs can induce the NALFD-like symptoms in cells as well as in the T2DM animal model. Hence, we hypothesized that targeting AGEs (MGO/GO-AGEs) or AGEs breaker might control the symptoms and severity of NAFLD in T2DM animals.

Among 13 synthesized naphthalenacyl thiazolium salts (KHAG series), KHAG-04, 05, 06, 11, 12, and 13 showed good AGEs breaking activities. These six KHAG compounds have a 1,4-dialkoxy group in the naphthalene ring, which may be one of the pharmacophoric moieties. Especially, KHAG-04 containing a 1,4-dimethoxynaphthalene ring could be the best candidate for AGEs breaker. A novel synthetic compound KHAG with a 1,4-dialkoxynaphthalene ring in the aryl acyl thiazolium structure dramatically induced the breakdown of MGO/GO-AGEs. The induction of macrophage-mediated pro-inflammation is responsible for the comorbidities in NAFLD and T2DM (Burgess et al., 2008). In this study, we observed that treatment of KHAG-04 inhibited the production of inflammatory mediators (NO) and pro-inflammatory cytokines (IL-1β and TNF-α) in inactivated macrophage cells without altering the cell viability and the level of IL-6 in LPS-activated microglia. Macrophage activation by MGO-AGEs or LPS was similar to a reported study (Subedi et al., 2020). A previous study revealed that an increased level of MGO/GO AGEs is responsible for the induction of inflammation in macrophage cells (Wetzels et al., 2017). Moreover, we found that MGO-AGEs strongly induced nitrite production in macrophages which was ameliorated by KHAG-04. Altogether, inhibition of the inflammatory mediators by KHAG-04 against LPS activated macrophages gave a possibility to expect a similar effect against MGO/GO-AGEs induced inflammatory conditions. Another interesting fact is that induction of AGEs breakdown by KHAG-04 can lower the level of total AGEs. Less amount of AGEs can induce comparatively less inflammation. Next, we assessed the effects of KHAG-04 in FFA-induced hepatic steatosis. KHAG-04 prominently decreased lipid accumulation and lipid metabolism–related markers such as FAS, SREBP1c, and C/EBPα in protein and RNA levels in hepatocytes. In addition, we reported here the MGO-AGEs capability to induce lipid accumulation and lipid metabolism–related markers such as FAS, and SREBP1c. Hence, the effects of KHAG-04 were analyzed on these factors related to lipogenesis. As expected, KHAG-04 prominently decreased lipid accumulation and lipid metabolism–related markers such as FAS, SREBP1c, and C/EBPα at the protein level.

Inhibition of the lipid/fat accumulation and blood glucose level is very crucial in the management of the NAFLD in T2DM (Xia et al., 2018). Our study supports the fact that NAFLD/T2DM condition showed a higher level of Fat/lipid together with increased blood glucose level; however, treatment of KHAG-04 inhibited the level of lipid concentration and lipid droplets together with the blood glucose level in OGTT and ITT condition in cells and animal model, respectively. Inhibition of the fatty acid synthesis and increase in fatty acid oxidation are the possible ways for lowering the level of lipid concentration in cells and lipid droplets in animal tissues. Acetyl-CoA carboxylase (ACC) is a key enzyme involved in the fatty acid synthesis, and it could be a great target for the treatment of NAFLD/T2DM (Goedeke et al., 2018). Our study supports this matter where we observed that KHAG-04 treatment reduced lipid/fat levels in cells and tissue along with the induction of ACC protein phosphorylation in comparison to the DMC group. A study suggested that inhibition of the ACC reverses the hepatic insulin resistance and NAFLD by upregulation of the triglyceride level in rodent animal models (Xia et al., 2018). Another research group revealed that food-induced liver steatosis was reversed by ACC inhibition by antisense oligonucleotide (Savage et al., 2006). In our study, we observed that the DMC and KHAG-04 treated animals showed a clear inhibition of phosphorylated ACC in comparison to the control group however the overall ratio of pACC/ACC was reversed by KHAG-04 treatment in comparison to the DMC animal group. At the same time, the KHAG-04 treatment significantly inhibited the expression of FAS, SREBP1c, and C/EBPα in comparison to the DMC group. All these proteins are responsible for fatty acid synthesis (Matsumoto et al., 2020) and inhibition of these proteins following KHAG-04 treatment supports the inhibition of fat/lipid accumulation. Inhibition of the fat or lipid accumulation can also be correlated with the reduced AGEs in fats or lipids are the sources of AGEs too. This might be the reason for the inhibition of lipid accumulation in animals treated with KHAG-04. In addition to this significant inhibition in the expression of the protein, inflammatory biomarkers were also observed in the animal tissue. Inhibition of the GO or MGO/GO-AGEs could be a reason for the inhibition of these inflammatory mediators including CRP, IL-6, and TNF-α. Subedi et al. presented that MGO and GO-AGEs have the capacity to induce inflammation through microglial activation (Subedi et al., 2020). Microglia and macrophage share a similar mechanism for the activation and production of inflammatory biomarkers because microglia are resident immune cells of the central nervous system (CNS) which is a type of macrophage (Perry and Teeling, 2013). From these studies, MGO/GO-AGEs in the DMC model might be able to increase macrophage activation and subsequent inflammation in the liver. However, inhibition of the liver GO level, as well as increased MGO/GO-AGEs breakdown, reduced the extent of inflammation in the KHAG-04 treated group and that is significant enough to lower the DMC mediated toxicity to animal models. Inconsistent with our *in vitro* and *in vivo* results, molecular docking results also showed that KHAG-04 has the highest binding affinity to lipogenic marker proteins than our positive control, metformin.

Overall, KHAG-04 strongly breakdown down MGO/GO-induced AGEs. It significantly reduced both LPS and MGO-AGEs stimulated nitrite production in RAW 264.7 cells and also decreased LPS-induced inflammatory cytokines. Furthermore, the KHAG-04 treatment group contained lower lipid droplets and also attenuates lipogenesis-related markers as compared to FFA and MGO-AGE– induced group in HepG2 cells. Treatment of KHAG-04 in the model of NAFLD of T2DM animals significantly inhibited lipid accumulation, fatty acid synthesis, and inflammatory marker in the liver, and these conditions seem to be possible through the inhibition of the blood glucose level together with its potency to induce the MGO/GO breakdown. Altogether, our newly synthesized KHAG-04 compound might be a potential therapeutic agent in terms of treating NAFLD in T2DM patients.

## Data Availability

The original contributions presented in the study are included in the article/Supplementary Material; further inquiries can be directed to the corresponding authors.

## Abbreviations

T2DM, type-2 diabetes mellitus; NAFLD, non-alcoholic fatty liver disease; AGEs, advanced glycation end products; ROS, reactive oxygen species; MGO, methylglyoxal; GO, glyoxal; IL-6, interleukin-6; IL-1β, interleukin-1β; TNF-α, tumor necrosis factor-α; SREBP1c, sterol regulatory binding protein 1c; FAS, fatty acid synthase; C/EBPα, CCAAT/enhancer-binding proteins; CRP, C-reactive protein; OGTT, The Oral Glucose Tolerance Test; ITT, Insulin Tolerance Test.
