# Supplementary material for: Identification of a potent NAFLD drug candidate for controlling T2DM-mediated inflammation and secondary damage in vitro and in vivo
Source: Front Pharmacol. 2022 Aug 19;13:943879. doi: 10.3389/fphar.2022.943879 (PMC9437277; doi:10.3389/fphar.2022.943879)

**Supporting information for**

**Identification of a potent NAFLD drug candidate for controlling the T2DM-mediated inflammation and secondary damage *in vitro* and *in vivo***

**Synthesis of KHAG compounds**

Synthesis of 4,5-dimethyl-3-(2-(naphthalen-2-yl)-2-oxoethyl) thiazol-3-ium bromide (KHAG-01).

2-(Bromoacetyl) naphthalene (1a; 0.5 g, 2.00 mmol) and 4,5-dimethylthiazole (0.28 mL, 4.01 mmol) were dissolved in acetonitrile (ACN; 5mL). The reaction mixture was refluxed for 12 h. After completion of the reaction by TLC, it was cooled to room temperature, filtered and crystallized in ACN to give a product KHAG-01 as an ivory solid.; Yield, 88%: ^1^H NMR (300MHz, (CD_3_)_2_SO) *δ* 9.99 (s, 1H), 8.82 (s, 1H), 8.20 (d, 1H, *J*= 8.0 Hz), 8.15 (d, 1H, *J*= 8.6 Hz), 8.08 (d, 1H, *J*= 7.6 Hz), 8.03 (d, 1H, *J*= 8.6 Hz), 7.79-7.68 (m, 2H), 6.51 (s, 2H), 2.56 (s, 3H), 2.36 (s, 3H): ^13^C NMR (75MHz, (CD_3_)_2_SO) *δ* 190.79, 157.98, 142.34, 135.59, 132.75, 131.92, 130.94, 130.81, 129.67, 129.46, 128.74, 127.88, 127.44, 123.24, 59.21, 12.00, 10.94: m.p. 234.3-236.5℃ decomposed: MS (EI, M^+^) for C_17_H_16_NOS^+^ calcd, 282.1; found, 282.0.

Synthesis of 3-(2-(1-Methoxynaphthalen-2-yl)-2-oxoethyl)-4,5-dimethylthiazol-3-ium bromide (KHAG-02).

2-Bromo-1-(1-methoxynaphthalen-2-yl) ethenone (1b; 0.3 g, 1.07 mmol) and 4,5-dimethylthiazole (0.15 mL, 162 mg, 2.2 mmol) were dissolved in ACN (5 mL). The reaction mixture was heated under reflux for 12 h. After checking the reaction complete by TLC, it was cooled to room temperature, concentrated in vacuo, filtered and washed with ethyl acetate (EA). The filtered was recrystallized in ACN and dried to give a product KHAG-02 as an ivory solid.; Yield, 51%: ^1^H NMR (300MHz, (CD_3_)_2_SO) *δ* 10.03 (s, 1H), 8.31 (d, 1H, *J*= 7.8Hz), 8.08 (d, 1H, *J*= 7.6Hz), 7.87 (s, 2H), 7.77-7.75 (m, 2H), 6.25 (s, 2H), 4.18 (s, 3H), 2.56 (s, 3H), 2.27 (s, 3H): ^13^C NMR (75MHz, (CD_3_)_2_SO) *δ* 190.68, 159.08, 158.07, 142.45, 142.26, 137.36, 132.51, 129.58, 128.39, 127.36, 127.13, 124.57, 123.72, 123.44, 64.37, 61.95, 11.95, 10.86

Synthesis of (3-(2-(1-hydroxy-4-methoxynaphthalen-2-yl)-2-oxoethyl)-4,5-dimethylthiazol-3-ium bromide)- KHAG-03.

Copper (II) bromide (1.08 g, 4.86 mmol) was added to ethanol (20 mL) and stirred at 78 ° C. After 15 min, 1- (1-hydroxy-4-methoxy naphthalen-2-yl) ethanone (2a; 0.7 g, 3.24 mmol) was added and stirred for an additional 12 hours at this temperature. If the reaction was not complete, further copper (II) bromide was added. After checking the reaction complete by TLC, the mixture was filtered through celite and concentrated in vacuo. It was purified by flash column chromatography (EA:Hexane = 1:5) to give the product 3a (2-bromo-1-(1-hydroxy-4-methoxynaphthalen-2-yl) ethanone) as a brown solid. (Yield: 40-50%): ^1^H NMR (300MHz, CDCl_3_) *δ* 8.30 (1H, d*, J*=8.3 Hz), 8.20 (1H, d, *J*=7.9 Hz), 7.74 (1H, t, *J*=7.7 Hz), 7.65 (1H, t, *J*=7.3 Hz), 6.82 (s, 1H), 4.82 (s, 2H), 3.99 (s, 3H)

4,5-dimethylthiazole (0.078 mL, 0.74 mmol) was added to the acyl bromide 3a (0.2 g, 0.67 mmol) in ACN (4 mL) . It was heated under reflux for 6 h. After checking the reaction complete by TLC, it was dissolved in ACN and cooled to room temperature. Then, it was recrystallized in ACN and EA, and dried to give a product KHAG-03 as a yellow solid. (Yield : 65%) ^1^H NMR (300MHz, CDCl_3_) *δ* 9.99 (s, 1H), 8.39 (d, 1H, *J*=9.0 Hz), 8.12-8.10 (m, 1H), 7.76-7.72 (m, 1H), 7.66-7.60 (m, 1H), 7.11 (s, 1H), 7.10 (s, 1H), 6.38 (s, 2H), 3.97 (s, 3H), 2.56 (s, 3H), 2.34 (s, 3H). ^13^C NMR (75MHz, (CD_3_)_2_SO) *δ* 194.07, 158.18, 154.87, 147.51, 142.42, 132.67, 130.39, 129.58, 127.23, 125.28, 124.13, 121.80, 111.93, 100.52, 60.00, 55.95, 11.96, 10.94: m.p. 116.2 ℃ decomposed: MS (EI, M^+)^ for C_18_H_8_NO_3_S^+^calcd, 328.1; found, 328.0.

Synthesis of 3-(2-(1,4-dimethoxynaphthalen-2-yl)-2-oxoethyl)-4,5-dimethylthiazol-3-ium bromide (KHAG-04).

The synthesis of the following compounds follows the synthesis procedure of KHAG-03

2-Bromo-1-(1,4-dimethoxynaphthaln-2-yl) ethenone (3b) from compound 2b; A yellow solid: Yield, 40-50%: ^1^H NMR (300MHz, CDCl_3_) *δ* 8.30 (1H, d, *J*=8.3 Hz), 8.20 (1H, d, *J*=7.9 Hz), 7.74 (1H, t, *J*=7.7 Hz), 7.65 (1H, t, *J*=7.3 Hz), 6.82 (s, 1H), 4.82 (s, 2H), 3.99 (s, 3H): ^13^C NMR (75MHz, (CD_3_)_2_SO) *δ* 192.84, 152.08, 151.81, 129.39, 128.18, 127.28, 124.48, 123.22, 122.68, 102.15, 64.22, 55.72, 36.48, 36.45

3-(2-(1,4-Dimethoxynaphthalen-2-yl)-2-oxoethyl)-4,5-dimethylthiazol-3-ium bromide (KHAG-04) from compound 3b: A yellow solid: Yield, 75%: ^1^H NMR (300MHz, (CD_3_)_2_SO) *δ* 10.05 (s, 1H), 8.28-8.24 (m, 2H), 7.80-7.77 (m, 2H), 7.19 (s, 1H), 6.26 (s, 1H), 4.13 (s, 3H), 4.00 (s, 3H), 2.56 (s, 3H), 2.35 (s, 3H): ^13^C NMR (75MHz, (CD_3_)_2_SO) *δ* 190.5, 158.1, 153.1, 151.4, 142.3, 132.5, 129.3, 129.1, 127.9, 123.9, 123.2, 122.3, 101.1, 64.3, 64.3, 62.1, 55.9, 12.0, 10.9: mp. 139.9-140.5 ℃ decomposed: MS (EI, M^+^) for C_19_H_20_NO_3_S^+^ calcd, 342.1; found, 342.1.

Synthesis of 3-(2-(5,8-dimethoxy-6-methylnaphthalen-2-yl)-2-oxoethyl)-4,5-dimethylthiazol-3-ium chloride (KHAG-05)

2-Chloro-1-(5,8-dimethoxy-6-methylnaphthalen-2-yl)ethenone (5); Aluminium chloride (1 g, 7.41 mmol) and chloroacetyl chloride (0.45 mL, 0.64 g, 5.65 mmol) in dry dichloromethane (DMC, 10 mL) were stirred under an argon atmosphere for 10 min. Then, 1,4-dimethoxy-2-methylnaphthalene (4; 1 g, 4.94 mmol) was added slowly and the reaction mixture was stirred for 18 h. Reaction was quenched with 1M aqueous hydrogen chloride. After 20 min, water was poured and the mixture was extracted with DMC. The organic layer was washed with 1N sodium bicarbonate, brine, and then dried over anhydrous Na_2_SO_4_ and purified by flash column chromatography (EA:Hexane= 1: 5) to give the product 5 as a yellow solid.; Yield, 44%: ^1^H NMR (300MHz, CDCl_3_) *δ* 8.81 (s, 1H), 8.03-8.11 (m, 2H), 6.68 (s, 1H), 4.88 (s, 2H), 4.00 (s, 3H), 3.86 (s, 3H), 2.48 (s, 3H): ^13^C NMR (75 MHz, CDCl_3_) *δ*: 190.87, 152.43, 146.86, 130.97, 130.37, 130.13, 124.94, 124.48, 123.93, 122.43, 107.88, 61.34, 55.65, 46.26, 16.60

The chloroacetyl intermediate 5 (0.3 g, 1.07 mmol) and 4,5-dimethylthiazole (0.15 mL, 2.14 mmol) were dissolved in ACN (4 mL). The reaction mixture was heated under reflux for 12 h. After cooling to room temperature, it was filtered and recrystallized in ACN to give the product KHAG-05 as a yellow solid.; Yield, 48 %: ^1^H NMR (300MHz, (CD_3_)_2_SO) *δ* 9.97 (s, 1H), 8.86 (s, 1H), 8.14 (d, 1H, *J*= 8.1Hz), 8.01 (d, 1H, *J*= 8.1 Hz), 7.03 (s, 1H), 6.57 (s, 2H), 4.03 (s, 3H), 3.81 (s, 3H), 2.56 (s, 3H), 2.46 (s, 3H), 2.34 (s, 3H): ^13^C NMR (75MHz, (CD_3_)_2_SO) *δ* 190.61, 158.05, 151.95, 146.38, 142.42, 132.63, 130.86, 130.45, 129.53, 124.75, 124.16, 123.21, 122.29, 108.91, 61.10, 59.19, 55.95, 16.32, 11.96, 10.83: mp. 199.8-200 ℃ decomposed: MS (EI, M^+^) for C_20_H_22_NO_3_S^+^ calcd, 356.1; found, 356.0.

Synthesis of 3-(2-(1,4-dimethoxy-3-methylnaphthalen-2-yl)-2-oxoethyl)-4,5-dimethylthiazol-3-ium bromide (KHAG-06)

1-(1,4-Dimethoxy-3-methylnaphthalen-2-yl) ethanone (2c; 0.58 g, 2.3 mmol) was dissolved in DMC (25 mL) and methanol (10 mL). Tetrabutylammonium tribromide (TBA-Br_3_, 0.84 g, 2.6 mmol) was added into the solution, stirred under argon atmosphere for 3h. Water was added into the mixture, extracted by dichloromethane, washed with water, 1M sodium bicarbonate and brine. The organic phase was dried over anhydrous Na_2_SO_4_ and purified by flash column chromatography (EA :Hexane = 1:5 ) to give the product 3c as a yellow solid. (Yield: 48%).; ^1^H NMR (300MHz, CDCl_3_) *δ* 8.11 (d, 1H, *J*= 8.0Hz), 8.06 (d, 1H, *J*= 8.0Hz), 7.61-7.53 (m, 2H), 4.50 (s, 2H), 3.89 (s, 3H), 3.88 (s, 3H), 2.34 (s, 3H): ^13^C NMR (75 MHz, CDCl_3_) *δ* 197.29, 150.71, 150.08, 129.87, 129.53, 127.77, 126.78, 126.27, 123.58, 122.50, 77.21, 63.97, 61.49, 36.88, 12.74

2-Bromo-1-(1,4-dimethoxy-3-methylnaphthalen-2-yl) ethanone (3c; 0.36 g, 1.1 mmol) and 4,5-dimethylthiazole (0.24 mL, 2.2 mmol) were dissolved in ACN (10 mL). The reaction mixture was heated under reflux for 12 h. After cooling to room temperature, the reaction mixture was concentrated and the residue was filtered. It was purified by the recrystalization in EA to give a product KHAG-06 as a yellow solid.; Yield, 52 %: ^1^H NMR (300MHz, (CD_3_)_2_SO) *δ* 10.13 (s, 1H), 8.14 (d, 2H, *J*=8.6Hz), 8.11 (d, 1H, *J*= 8.0Hz) 7.76-7.68 (m, 2H), 6.18 (s, 2H), 3.93 (s, 3H), 3.85 (s, 3H), 2.72 (s, 3H), 2.45 (s, 3H), 2.32 (s, 3H): ^13^C NMR (75MHz, (CD_3_)_2_SO) *δ* 197.07, 158.03, 150.39, 150.32, 142.05, 133.25, 129.58, 128.55, 127.87, 126.87, 126.03, 122.99, 122.64, 122.39, 64.47, 62.55, 61.47, 12.57, 12.05, 10.83: mp. 181.4-182.7 ℃ decomposed: MS (EI, M^+^) for C_20_H_22_NO_3_S^+^ calcd, 356.1; found, 356.0.

General synthesis of 3-(2-(4-acetoxy-1-methoxynaphthalen-2-yl)-2-oxoethyl)-4,5-dimethylthiazol-3-ium bromide (KHAG-07)

3-(2-Bromoacetyl)-4-methoxynaphthalen-1-yl (7a); To a solution of acetyl intermediate 6a (0.26 g, 0.9 mmol) in DMC (20 mL), TBA-Br_3_ (0.63 g, 1.9 mmol) was added and stirred under an argon atmosphere for 3 h. The reaction was quenched with water, extracted by DMC, and washed with water, 1M sodium bicarbonate and brine. The combined organic layers were dried over anhydrous Na_2_SO_4_ and concentrated in vacuo. The crude product was purified by flash column chromatography (EA:Hexane = 1:5) to yield 3-(2-bromoacetyl)-4-methoxynaphthalen-1-yl (7a) as a yellow solid.; Yield, 47 %: ^1^H NMR (300MHz, CDCl_3_) *δ* 8.23-8.12 (m, 1H), 7.87-7.82 (m, 1H), 7.76-7.63 (m, 2H), 7.54 (s, 1H), 4.74 (s, 2H), 4.01 (s, 3H), 2.46 (s, 3H): ^13^C NMR (75 MHz, CDCl_3_) *δ* 191.76, 169.33, 155.80, 143.21, 130.65, 129.36, 128.70, 127.44, 124.58, 123.96, 121.99, 117.71, 64.56, 36.13, 20.91

Bromoacetyl intermediate 7a (0.15 g, 0.4 mmol) and 4,5-dimethylthiazole (0.09 mL, 0.8 mmol) were dissolved in ACN (5 mL). The reaction mixture was heated under reflux for 12 h. After cooling to room temperature and evaporating solvent, the residue was recrystallized from EA. Then, the solid was filtered, washed with EA and dried to yield the product KHAG-07 as an ivory solid.; Yield, 20%: ^1^H NMR (300MHz, (CD_3_)_2_SO) *δ* 10.01 (s, 1H), 8.35 (d, 1H, *J*= 9.7Hz), 8.03 (d, 1H, *J*= 8.7Hz), 7.85-7.80 (m, 2H), 6.24 (s, 2H), 4.20 (s, 3H), 2.56 (s, 3H), 2.47 (s, 3H), 2.35 (s, 3H): ^13^C NMR (75MHz, (CD_3_)_2_SO) *δ* 189.86, 169.53, 158.13, 156.96, 142.91, 142.36, 132.49, 130.69, 130.27, 128.17, 128.03, 124.32, 123.21, 122.30, 116.57, 64.52, 61.91, 20.71, 11.95, 10.85: mp. 166.8-167 ℃ decomposed: MS (EI, M^+^) for C_20_H_20_NO_4_S^+^ calcd, 370.1; found, 370.0.

The following synthesis for each KHAG-08, KHAG-09, and KHAG-10 was performed in the same manner as for KHAG-07, except that different alkylating agents, such as 2-bromopropane for KHAG-08, isoamyl bromide for KHAG-09, and 2-methoxyethoxy tosylate for KHAG-10, were used instead of iodomethane.

Synthesis of 3-(2-(4-acetoxy-1-isopropoxynaphthalen-2-yl)-2-oxoethyl)-4,5-dimethylthiazol-3-ium (KHAG-08)

3-(2-Bromoacetyl)-4-isopropoxynaphthalen-1-yl acetate (**7b**); Yield, 35% as a brown oil: ^1^H NMR (300MHz, CDCl_3_) *δ* 8.18 (d, 1H, *J*=6.7 Hz), 7.84 (d, 1H, *J*= 7.8 Hz), 7.67-7.58 (m, 2H), 7.40 (s, 1H), 4.77 (s, 2H), 4.41-4.32 (m, 1H), 2.43 (s, 3H), 1.35 (d, 6H, *J*=6.0 Hz): ^13^C NMR (75 MHz, CDCl_3_) *δ* 194.33, 169.29, 152.51, 142.87, 130.19, 129.68, 129.02, 127.10, 126.56, 124.22, 121.83, 117.49, 80.00, 35.66, 22.43, 20.94

3-(2-(4-Acetoxy-1-isopropoxynaphthalen-2-yl)-2-oxoethyl)-4,5-dimethylthiazol-3-ium bromide (KHAG-08)*;* Yield, 75% as an ivory solid: ^1^H NMR (300MHz, (CD_3_)_2_SO) *δ* 10.04 (s, 1H), 8.30 (d, 1H, *J*= 8.7 Hz ), 8.01 (d, 1H, *J*= 7.1 Hz), 7.84-7.74 (m, 2H), 7.60 (s, 1H), 6.22 (s, 2H), 4.63-4.58 (m, 1H), 2.56 (s, 3H), 2.35 (s, 3H), 2.28 (s, 3H), 1.39 (d, 6H, *J*=6.0 Hz): ^13^C NMR (75MHz, (CD_3_)_2_SO) *δ* 191.39, 169.46, 158.23, 153.58, 142.42, 142.10, 132.61, 130.24, 129.97, 129.04, 127.65, 124.73, 124.57, 122.04, 116.77, 80.12, 61.59, 21.87, 20.72, 12.00, 10.99: mp. 199.7-199.9 ℃ decomposed: MS (EI, M^+^) for C_22_H_24_NO_4_S^+^ calcd, 398.1; found, 398.1.

Synthesis of 3-(2-(4-acetoxy-1-(isopentyloxy) naphthalen-2-yl)-2-oxoethyl)-4,5-dimethylthiazol-3-ium bromide (KHAG-09)

3-(2-Bromoacetyl)-4-(isopentyloxy) naphthalen-1-yl(7c); Yield, 26% as a yellow liquid: ^1^H NMR (300MHz, CDCl_3_) *δ* 8.19 (d, 1H, *J*= 7.3 Hz), 7.84 (d, 1H, *J*= 7.1 Hz), 7.67-7.61 (m, 2H), 7.48 (s, 1H), 4.75 (s, 2H), 4.07 (t, 2H, *J*=6.8 Hz), 2.46 (s, 3H), 1.88-1.85 (m, 3H), 1.05 (d, 6H, *J*= 6.0 Hz): ^13^C NMR (75 MHz, CDCl_3_) *δ* 192.60, 169.31, 154.74, 142.99, 130.43, 129.21, 129.03, 127.33, 125.04, 123.79, 121.88, 117.62, 76.85, 39.00, 35.68, 25.05, 22.65, 20.89

3-(2-(4-Acetoxy-1-(isopentyloxy)naphthalen-2-yl)-2-oxoethyl)-4,5-dimethylthiazol-3-ium bromide (KHAG-09); Yield, 85% as an ivory solid: ^1^H NMR (300MHz, (CD_3_)_2_SO) *δ* 10.00 (s, 1H), 8.28-8.24 (m, 1H), 8.03-8.01 (m, 1H), 7.83-7.80 (m, 2H), 7.67 (s, 1H), 6.19 (s, 2H), 4.26 (t, 2H, *J*=3.0 Hz), 2.56 (s, 3H), 2.35 (s, 3H), 1.99-1.90 (m, 3H), 1.05 (d, 6H, *J*= 6.0Hz): ^13^C NMR (75MHz, (CD_3_)_2_SO) *δ* 189.92, 169.54, 158.12, 155.65, 142.78, 142.33, 132.48, 130.57, 130.22, 128.44, 128.08, 123.91, 123.47, 122.30, 116.72, 76.25, 61.57, 38.31, 24.62, 22.59, 20.71, 11.95, 10.90: m.p. 170.2-170.8 ℃ decomposed: MS (EI, M^+^) for C_24_H_28_NO_4_S^+^ calcd, 426.1; found, 426.1.

Synthesis of 3-(2-(4-acetoxy-1-(2-methoxyethoxy) naphthalen-2-yl)-2-oxoethyl)-4,5-dimethylthiazol-3-ium (KHAG-10)

3-(2-Bromoacetyl)-4-(2-methoxyethoxy) naphthalen-1-yl acetate (**7d**); Yield, 30% as a yellow liquid: ^1^H NMR (300MHz, CDCl_3_) *δ* 8.28-8.25(m, 1H), 7.91-7.79(m, 1H), 7.78-7.61(m, 2H), 7.60(s, 1H), 4.88(s, 2H), 4.26-4.23(m, 2H), 4.17-4.14(m, 2H), 3.45(s, 3H), 2.46(s, 3H): 13C NMR (75 MHz, CDCl3) *δ*: 193.09, 169.71, 153.91, 143.71, 130.21, 129.59, 128.39, 127.84, 126.19, 124.36, 122.36, 118.15, 76.58, 71.38, 59.56, 37.18, 21.32.

3-(2-(4-Acetoxy-1-(2-methoxyethoxy)naphthalen-2-yl)-2-oxoethyl)-4,5-dimethylthiazol-3-ium (KHAG-10); Yield, 67% as a brown solid: ^1^H NMR (300MHz, (CD_3_)_2_SO) *δ* 9.98 (s, 1H), 8.42 (d, 1H, *J*= 8.4 Hz), 8.02 (d, 1H, *J*= 7.1 Hz) 7.86-7.77 (m, 2H), 7.62 (s, 1H), 6.30 (s, 2H), 4.44 (br s, 2H), 3.85 (br s, 2H), 3.38 (s, 3H), 3.26 (s, 3H), 2.56 (s, 3H), 2.47 (s, 3H), 2.36 (s, 3H): ^13^C NMR (75MHz, (CD_3_)_2_SO) δ 190.23, 169.53, 158.03, 155.08, 142.97, 142.38, 132.54, 130.57, 130.19, 128.27, 127.97, 124.24, 123.87, 122.23, 116.66, 76.14, 70.89, 61.86, 58.43, 20.72, 11.98, 10.82: mp. 116.2-119 ℃ decomposed: MS (EI, M^+^) for C_22_H_24_NO_5_S^+^ calcd, 414.1; found, 414.1.

2.2.11. Synthesis of 3-(2-(1,4-diisopropoxynaphthalen-2-yl)-2-oxoethyl)-4,5-dimethylthiazol-3-ium bromide (KHAG-11)

1-(1,4-Diisopropoxynaphthalen-2-yl)ethanone (8a); Compound 6b (0.12 g, 0.42 mmol) was dissolved in methanol (5 mL) and was cooled to 0 ℃. Then 1 wt% potassium hydroxide solution in methanol (1 mL) was added. The mixture was stirred for 2 h. The mixture was neutralized by adding Amberlite IR-120 (H) and stirred for an additional 15 min. Amberlite IR-120 (H) was then removed by filtration and the filtrate was concentrated in vacuo. The resulting crude intermediate was dissolved in DMF (3 mL), and 2-bromopropane (0.06 mL, 0.63 mmol) and cesium carbonate (0.38 g, 1.26 mmol) were added. The mixture was heated under reflux. After checking the reaction complete by TLC, it was cooled to room temperature. The reaction mixture was extracted with DMC and washed with water. It was concentrated in vacuo and purified by flash column chromatography (EA:Hexane = 1:5) to give the product 8a as a brown oil; Yield, 83%: ^1^H NMR (300MHz, CDCl_3_) *δ* 8.26 (d, 1H, *J*=8.7 Hz), 8.13 (d, 1H, *J*=9.0 Hz), 7.60-7.53 (m, 2H), 6.95 (s, 1H), 4.80-4.72 (m, 1H), 4.31-4.25 (m, 1H), 2.76 (s, 3H), 1.44 (d, 6H, *J*=6.0 Hz), 1.31(d, 6H, *J*=6.0 Hz): ^13^C NMR (75 MHz, CDCl_3_) *δ*:202.57, 149.65, 147.77, 130.01, 129.56, 129.47, 127.20, 126.59, 123.56, 122.69, 104.40, 78.83, 70.56, 30.80, 22.35, 22.11

2-Bromo-1-(1,4-diisopropoxynaphthalen-2-yl) ethanone (9a); Compound 8a(0.17 g, 0.63 mmol) was dissolved in DMC (10 mL). After adding TBA-Br_3_ (0.22 g, 0.7 mmol), it was stirred at room temperature under an argon atmosphere for 3 h. After the concentration in vacuo, water was added into the mixture and it was extracted with DMC, washed with water, 1M sodium bicarbonate and brine. The organic phase was dried over anhydrous Na_2_SO_4_ and concentrated in vacuo. The crude product was purified by flash column chromatography (EA:Hexane = 1:5) to give a pure product 9a.; Yield, 56%: ^1^H NMR (300MHz, CDCl_3_) *δ* 8.28 (d, 1H, *J*=9.0 Hz), 8.09 (d, 1H, *J*=6.7 Hz), 7.60-7.55 (m, 2H), 6.92 (s, 1H), 4.79 (s, 2H), 4.79-4.76 (m, 1H), 4.32-4.26 (m, 1H), 1.49 (d, 6H, *J*=9.1 Hz), 1.31 (d, 6H, *J*=9.0 Hz): ^13^C NMR (75 MHz, CDCl_3_) *δ* 195.71, 150.05, 147.77, 129.89, 129.44, 127.68, 126.90, 123.48, 122.92, 104.30, 100.53, 70.65, 36.02, 22.36, 22.03

Acyl bromide compound 9a (0.13 g, 0.35 mmol) and 4,5-dimethylthiazole (0.074 mL, 0.71 mmol) were dissolved in ACN (2 mL). The reaction mixture was heated under reflux for 12h. After cooling to room temperature, solvent was evaporated and the residue was filtered. It was purified by the recrystallization in EA to give a product KHAG-11 as an ivory solid; Yield, 55%: ^1^H NMR (300MHz, (CD_3_)_2_SO) *δ* 10.07 (s, 1H), 8.23-8.17 (m, 1H), 7.75-7.71 (m, 2H), 7.11 (s, 1H), 6.20 (s, 2H), 4.84-4.78 (m, 1H), 4.53-4.48 (m, 1H), 2.55 (s, 3H), 2.30 (s, 3H), 1.40 (d, 6H, *J*=6.0 Hz), 1.36 (d, 6H, *J*=6.0 Hz): ^13^C NMR (75MHz, (CD_3_)_2_SO) *δ* 192.27, 158.14, 149.13, 148.90, 142.05, 132.62, 129.58, 128.93, 128.72, 127.45, 125.27, 124.03, 122.39, 103.65, 79.19, 70.48, 61.76, 21.81, 21.72, 11.98, 10.98: mp. 187.8-188.6 ℃ decomposed: MS (EI, M^+^) for C_23_H_28_NO_3_S^+^ calcd, 398.2; found, 398.1.

Synthesis of 3-(2-(4-isopropoxy-1-methoxynaphthalen-2-yl)-2-oxoethyl)-4,5-dimethylthiazol-3-ium bromide (KHAG-12)

1-(4-Isopropoxy-1-methoxynaphthalen-2-yl) ethanone (8b) was prepared 3-acetyl-4-methoxynaphthalen-1-yl acetate (6a) in the same synthetic procedure as for 8a.; Yield, 51% as a yellow oil : ^1^H NMR (300MHz, CDCl_3_) *δ* 8.31-8.18 (m, 1H), 8.17-8.12 (m, 1H), 7.60-7.55 (m, 2H), 7.09 (s, 1H), 4.83-4.75 (m, 1H), 3.95 (s, 3H), 2.79 (s, 3H), 1.44 (d, 6H, *J*= 6.0 Hz): ^13^C NMR (75 MHz, CDCl_3_) *δ* 199.97, 151.41, 149.95, 130.02, 128.89, 127.58, 127.33, 126.97, 123.14, 122.91, 104.40, 70.55, 63.83, 30.98, 22.06

2-Bromo-1-(4-isopropoxy-1-methoxynaphthalen-2-yl) ethanone (9b); The same α-bromination of compound 8b by TBA-Br_3_ gave the corresponding acyl bromide compound 9b; Yield, 70% as a yellow oil: ^1^H NMR (300MHz, CDCl_3_) *δ* 8.33-8.27 (m, 1H), 8.16-8.10 (m, 1H), 7.63-7.57 (m, 2H), 7.07 (s, 1H), 4.78 (s, 2H), 3.97 (s, 3H), 1.45 (d, 6H, *J*= 6.0 Hz): ^13^C NMR (75 MHz, CDCl_3_) *δ* 192.88, 151.38, 150.25, 130.32, 128.35, 128.01, 127.17, 124.53, 123.15, 123.02, 104.25, 70.58, 64.21, 36.69, 21.98, 21.92

The reaction of compound 9b with 4,5-dimethylthiazole yielded a thiazolium salt KHAG-12.; Yield, 68% as an ivory solid: ^1^H NMR (300MHz, (CD_3_)_2_SO) *δ* 10.03 (s, 1H), 8.25 (d, 1H, *J*= 3.3 Hz), 8.23 (d, 1H, *J*= 3.2 Hz), 7.79-7.75 (m, 2H), 7.19 (s, 1H), 6.24 (s, 2H), 4.85-4.76 (m, 1H), 4.12 (s, 3H), 2.56 (s, 3H), 2.34 (s, 3H), 1.40 (d, 6H, *J*= 6.0 Hz): ^13^C NMR (75MHz, (CD_3_)_2_SO) *δ* 190.56, 158.08, 152.66, 149.45, 142.28, 132.49, 130.03, 129.07, 128.08, 127.83, 123.79, 123.27, 122.59, 103.34, 70.50, 64.27, 62.01, 21.68, 11.96, 10.89: mp. 99.3-100.8 ℃ decomposed: MS (EI, M^+^) for C_21_H_24_NO_3_S^+^ calcd, 370.1; found, 370.1.

Synthesis of 3-(2-(4-(allyloxy)-1-methoxynaphthalen-2-yl)-2-oxoethyl)-4,5-dimethylthiazol-3-ium bromide (KHAG-13)

1-(4-(Allyloxy)-1-methoxynaphthalen-2-yl)ethenone (8c); The synthesis for 8c was performed from compound 6a in the same manner as for 8a, except that a different alkylating agent, ally bromide.; Yield, 70% as a brown oil: ^1^H NMR (300MHz, CDCl_3_) *δ* 8.35-8.18 (m, 1H), 8.17-8.15 (m, 1H), 7.63-7.57 (m, 2H), 7.10 (s, 1H), 6.23-6.10 (m, 1H), 5.56-5.32 (m, 2H), 5.31-4.70 (m, 2H), 3.96 (s, 3H), 2.79 (s, 3H): ^13^C NMR (75 MHz, CDCl_3_) *δ* 199.80, 151.94, 150.64, 133.02, 129.23, 128.80, 127.81, 127.18, 127.11, 123.24, 122.67, 117.63, 103.3369.13, 63.86, 30.94

1-(4-(Allyloxy)-1-methoxynaphthalen-2-yl)-2-bromoethanone 9c); The same α-bromination of compound 8c by TBA-Br_3_ gave a corresponding acyl bromide compound 9c.; Yield, 70% as a yellow oil: ^1^H NMR (300MHz, CDCl_3_) *δ* 8.35-8.31 (m, 1H), 8.16-8.13 (m, 1H), 7.68-7.61 (m, 2H), 7.07 (s, 1H), 6.23-6.10 (m, 1H), 5.56-5.49 (m, 1H), 5.37-5.33 (m, 1H), 4.77 (s, 2H), 3.98 (s, 3H): ^13^C NMR (75 MHz, CDCl_3_) *δ*: 192.81, 151.94, 150.97, 132.77, 129.60, 128.27, 127.34, 124.42, 123.27, 122.81, 117.80, 103.34, 69.15, 64.29, 36.62, 20.63

The reaction of compound 9c with 4,5-dimethylthiazole yielded a thiazolium salt KHAG-13.; Yield, 54% as an ivory solid: ^1^H NMR (300MHz, (CD_3_)_2_SO) *δ* 10.04 (s, 1H), 8.32-8.25 (m, 2H), 7.81-7.77 (m, 2H), 7.12 (s, 1H), 6.24 (s, 2H), 6.22-6.10 (m, 1H), 5.56-5.55 (m, 1H), 5.50-5.32 (m, 2H), 4.13 (s, 3H), 2.56 (s, 3H), 2.34 (s, 3H): ^13^C NMR (75MHz, (CD_3_)_2_SO) *δ* 190.43, 158.08, 153.09, 150.11, 142.24, 133.23, 132.49, 132.47, 129.24, 127.93, 127.89, 123.86, 123.15, 122.32, 117.53, 102.48, 68.76, 64.33, 62.02, 11.95, 10.88: mp. 161.7-163 ℃ decomposed: MS (EI, M^+^) for C_21_H_22_NO_3_S^+^ calcd, 368.1; found, 368.1.

**Supplementary table 1**

**Table S1. Effect of 13 compounds GO-AGEs breakdown.**

| Sample | Free amines (%) |
| --- | --- |
| GO-AGEs (1 mg/ml) | 0.00 ± 1.85 |
| KHAG-01 | 16.74 ± 1.24 |
| KHAG-02 | 14.48 ± 0.91 |
| KHAG-03 | -1.78 ± 2.86 |
| KHAG-04 | 32.19 ± 3.93^***^ |
| KHAG-05 | 21.04 ± 8.81^***^ |
| KHAG-06 | 13.44 ± 1.27 |
| KHAG-07 | 11.08 ± 0.93 |
| KHAG-08 | 12.39 ± 2.50 |
| KHAG-09 | 2.84 ± 0.70 |
| KHAG-10 | 14.25 ± 2.28 |
| KHAG-11 | 17.51 ± 1.97 |
| KHAG-12 | 20.73 ± 3.15 |
| KHAG-13 | 18.91 ± 0.65 |
| Aminoguanidine (1 mM) | 25.55 ± 0.96^***^ |

The GO-AGEs breaking ability of 15 compounds was measured by breaking MGO-BSA using the TNBSA assay. The percentage of each experiment is presented as the mean ± SEM of three independent experiments. (^***^p < 0.001 vs. GO-AGEs)

**Supplementary table 2**

**Table S2. Effect of 13 compounds MGO-AGEs breakdown.**

| Sample | Free amines (%) |
| --- | --- |
| MGO-AGEs (1 mg/ml) | 0.00 ± 1.51 |
| KHAG-01 | 18.39 ± 0.30 |
| KHAG-02 | 16.17 ± 2.53 |
| KHAG-03 | 2.29 ± 2.31 |
| KHAG-04 | 24.94 ± 4.89^***^ |
| KHAG-05 | 24.62 ± 4.69^***^ |
| KHAG-06 | 10.34 ± 0.44 |
| KHAG-07 | 12.79 ± 2.00 |
| KHAG-08 | 10.85 ± 2.62 |
| KHAG-09 | 7.94 ± 2.38 |
| KHAG-10 | 13.31 ± 1.23 |
| KHAG-11 | 18.65 ± 1.62 |
| KHAG-12 | 19.51 ± 0.20 |
| KHAG-13 | 20.58 ± 2.91 |
| Aminoguanidine (1 mM) | 22.03 ± 1.02^***^ |

The MGO-AGEs breaking ability of 15 compounds was measured by breaking MGO-BSA using the TNBSA assay. The percentage of each experiment is presented as the mean ± SEM of three independent experiments. (^***^p < 0.001 vs. MGO-AGEs)

**Supplementary figures**

**Figure S1. Effects of KHAG-05 on LPS-induced Nitrite production and pro-inflammatory cytokines (IL-1β and TNF-α) secretion in Raw 264.7 cells.** Raw 264.7 cells were pre-treated with 0.1, 1, and 10 μM of KHAG-05 for 1 h and stimulated with 100 ng/ml of LPS for an additional 23 h. (A) Nitrite production assay. (B) Cell viability on Raw 264.7 cells was measured using an MTT assay. (C, D) The secretion of IL-1β and TNF-α in the supernatant was evaluated following the manufacturer’s ELISA assay kit protocol. All data are indicated as mean ± SEM (###p < 0.001 vs. Control, *p < 0.05, ***p < 0.001 vs. 100 ng/ml LPS)


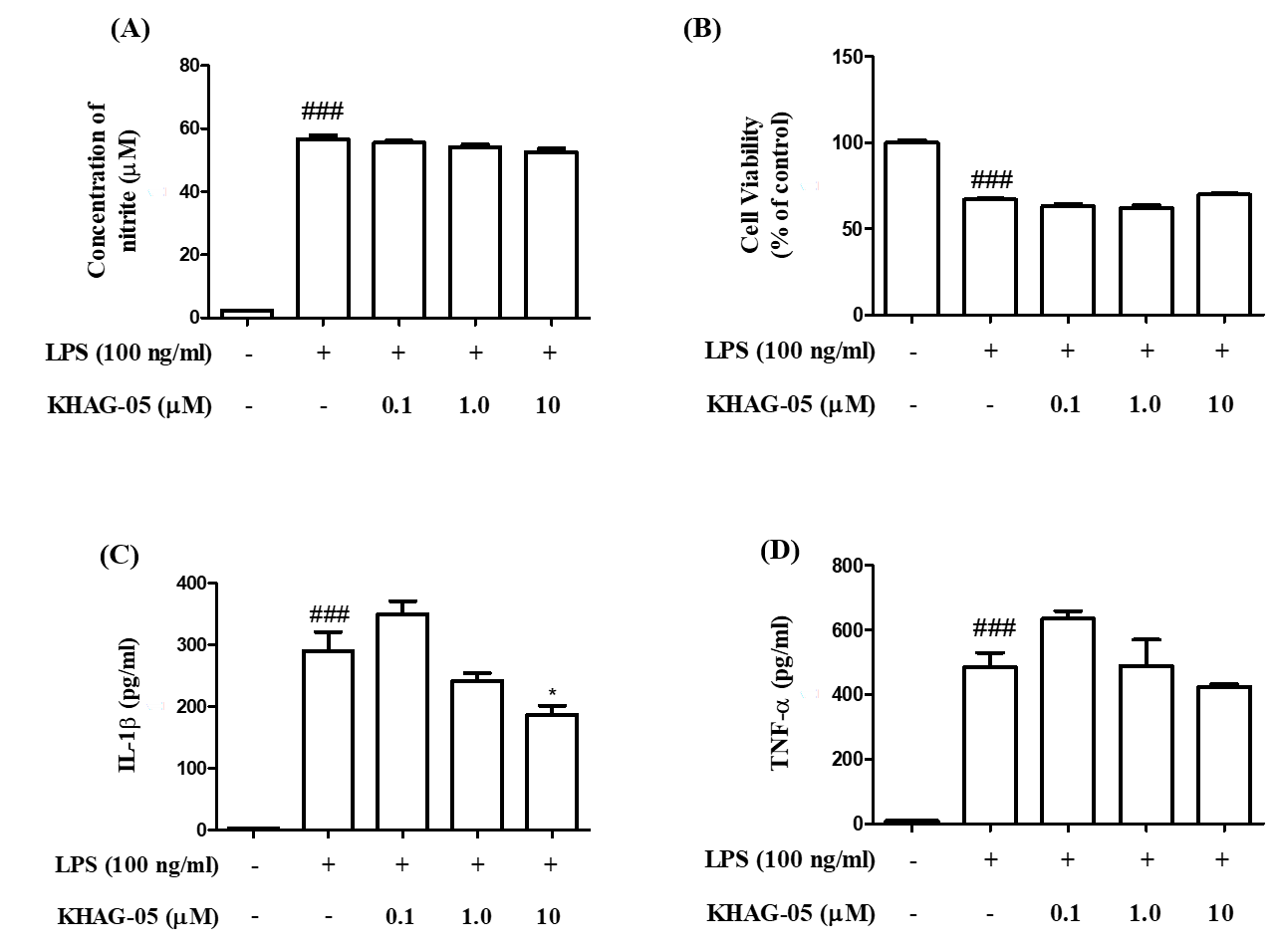


**Figure S2. Effects of KHAG-04 on GO levels in the livers of T2DM mice.** HPLC chromatograms. (A) A mixture of the standard compounds; 2-MQ. (B) A mixture of the standard compounds; Quinoxaline. (C) Chromatogram of normal mice. (D) Chromatogram of HFD+STZ-induced mice. (E) Chromatogram of HFD+STZ+KHAG 10 mg/kg mice.


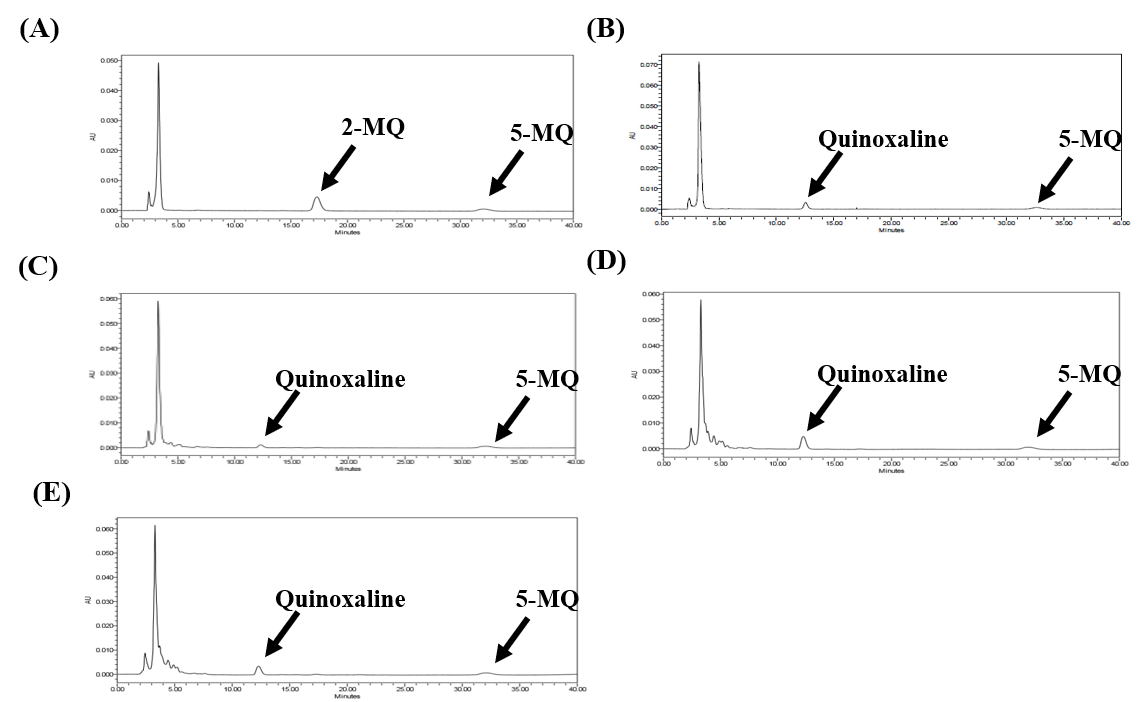

Supplement: Supplementary file 1 [file DataSheet1.DOCX]
